# Forecasting volatility in Asian financial markets: evidence from recursive and rolling window methods

**DOI:** 10.1007/s43546-022-00329-9

**Published:** 2022-09-29

**Authors:** Mehmet Sahiner

**Affiliations:** Division of Accounting and Finance, University of Stirling, Stirling, FK9 4LA UK

**Keywords:** Volatility, Forecasting, G(ARCH), Forecast evaluation, C22, C53, C58, G17

## Abstract

The present paper examines the relative out-of-sample predictive ability of GARCH, GARCH-M, EGARCH, TGARCH and PGARCH models for ten Asian markets by using three different time frames and two different methods, considering the features of volatility clustering, leverage effect and volatility persistence phenomena, for which the evidence of existence is found in the data. Five measures of comparison are employed in this research, and a further dimension is investigated based on the classification of the selected models, in order to identify the existence or lack of any differences between the recursive and rolling window methods. The empirical results reveal that asymmetric models, led by the EGARCH model, provide better forecasts compared to symmetric models in higher time frames. However, when it comes to lower time frames, symmetric GARCH models tend to outperform their asymmetric counterparts. Furthermore, linear GARCH models are penalized more by the rolling window method, while recursive method places them amongst the best performers, highlighting the importance of choosing a proper approach. In addition, this study reveals an important controversy: that one error statistic may suggest a particular model is the best, while another suggests the same model to be the worst, indicating that the performance of the model heavily depends on which loss function is used. Finally, it is proved that GARCH-type models can appropriately adapt to the volatility of Asian stock indices and provide a satisfactory degree of forecast accuracy in all selected time frames. These results are also supported by the Diebold-Mariano (DM) pairwise comparison test.

## Introduction

Volatility is the degree of variation of a trading price series over time, and is usually measured by the standard deviation of logarithmic returns. As an important concern for traders, investors, companies and financial regulatory authorities, volatility forecasts of asset returns have been studied over the years for risk management, security valuation, portfolio diversification and monetary policy making purposes. Furthermore, volatility modelling and forecasting have especially attracted finance professionals and academics following the stock market crash in 1987, since the main reason for the crash was attributed to high volatility (Haugen et al. 1991).

The behavior of stock market volatility is time-varying. The early prominent empirical works of Mandelbrot (1963) and Fama (1965) revealed that small (large) changes in asset prices tend to be followed by small (large) price changes of the same magnitude, a phenomenon known as volatility clustering. Throughout the empirical applications over the last five decades, evidence suggests that volatility changes of return series are predictable, particularly in the long-term (Fama and French 1989; Wurgler 2000; Cochrane 2008; Campbell and Thompson 2008). Therefore, numerous empirical models and methods have been developed and applied to identify and accurately predict the volatility behavior of return series. Nevertheless, earlier studies reveal no consensus regarding which model or method can provide the most accurate forecasts of asset returns.

Early studies tried to predict future volatility through simple statistical approaches based on averaging and smoothing methods. However, these simple models had limited prediction capacity, as financial time series tend to harbor certain special characteristics, such as volatility clustering. In order to deal with this issue, Engle (1982) developed the first generation of heteroscedasticity models with the seminal idea on ARCH models. Bollerslev (1986) took another step and put forward a generalized version, called the GARCH model. Although the ARCH and GARCH models received incredible attention from researchers and practitioners and proved their empirical success, these models were still not able to capture the stylized fact of volatility asymmetry, which was later named the leverage effect by Black (1976). This constraint has been solved by the development of more adaptable and advanced versions. Noteworthy and popular examples of this new model class are Nelson’s (1991) Exponential GARCH (EGARCH) model, Ding et al.’s (1993) Power GARCH (PGARCH) model, and Zakoian’s (1994) Threshold GARCH (TGARCH) model. A number of studies have been devoted to reviewing the important GARCH family models, such as Poon and Granger (2003), Bauwens et al. (2006), Silvennoinen and Teräsvirta (2009), and Bhowmik and Wang (2020).

The aim of the present paper is to investigate and evaluate the relative out-of-sample forecasting ability of linear and non-linear GARCH models by comparing daily, weekly, and monthly frequencies, using recursive and rolling window methods. However, evaluation of predicted models is not an easy task and one of the major issues is that the “true” volatility series is not observed. To overcome of this problem, the squared return series are used as a proxy for the unobserved volatility process, since squared returns are an unbiased gauge for volatility, as revealed by Andersen and Bollerslev (1998). With the usage of squared returns, proper evaluation of the estimated models is ensured in terms of selected error statistics.

Another important aspect of the paper is its coverage of a broad range of Asian markets, including those of emerging economies. Although there are a significant number of papers on forecasting stock market volatility, there are limited studies examining the Asian markets, particularly on emerging markets. The review of Poon and Granger (2003) reports only five out of 93 papers on volatility forecasting cover Asian markets, namely New Zealand, Australia and Japan, and none at all on emerging Asian markets. Some recent papers have individually examined stock market volatility in Asian markets, including Ibrahim et al. (2020) for Asia–Pacific markets, Pati et al. (2018) for India, Australia and Hong Kong, and Duan et al. (2021) for Taiwan. However, the stock markets of emerging countries such as Indonesia, Thailand, Malaysia, and the Philippines, which together constitute 66% of the market capitalization of the ASEAN economies as of 2016 (Ganbold 2021), tend to be ignored in volatility exercises. In addition, volatility dynamics in emerging stock markets of Asia is expected to influence the global stock markets through the “leverage effect” and idiosyncratic risk factors (Atanasov 2018; Bouri et al. 2020), and hence further indicating the importance of generating more accurate and comprehensive forecasts in these bloc. Therefore, this paper aims to extend the literature of volatility forecasting by selecting ten Asian markets with up-to-date data and covering periods of both financial crisis and recent developments.

It is broadly acknowledged by the financial literature that an increase in data frequency is accompanied by excess kurtosis, which challenges the capabilities of forecasting models due to the fat-tailed distribution on return series (Mandelbrot 1963). Under assumption of normality for errors, the results of the models would be biased. Therefore, the present paper considers student’s *t* distribution in all selected time frames to capture anomalies in the return series. Furthermore, it aims to contribute to the ongoing debate for determining the best model between linear (symmetric) and non-linear (asymmetric) GARCH family models for producing the most accurate volatility forecasts.

This research adds to the current academic literature in three ways. First, it finds that GARCH-type models can appropriately adapt to the volatility behaviour of Asian stock indices and provide a satisfactory degree of forecast accuracy in all selected time frames. The superiority of asymmetric models is more evident for higher time frames, while symmetric models tend to outperform asymmetric ones in lower time frames. Second, given the level of risk associated with investment in stock markets, day traders, investors, financial analysts, and empirical finance professionals should consider alternative error distributions while specifying a predictive volatility model, as less contributing error distributions implies incorrect specification, which could lead to loss of efficiency in the model. Investors should also not ignore the impact of news while forming expectations of investments. Finally, the obtained results report that frequency of data and choice of forecast method have a strong effect on the performance of the models, and therefore, depending on the investment perspective and risk sensitivity, correct method and time frames should be applied.

The remainder of the paper is organized as follows. Section 2 provides a literature review of volatility forecasting applications on various markets with the emphasis on Asian markets. Section 3 reports the methodology used and Sect. 4 provides the data. Section 5 presents the empirical analysis and results. Finally, Sect. 6 discusses the study findings and concludes.

## Literature review

Numerous studies in the existing literature have applied various approaches to the question of a superior forecasting model, yet a consensus still has not been reached. Since the stock market incidents in the early 1990s, triggered by the Japanese asset price bubble and Hong Kong’s stock market collapse in 1992, a significant amount of study has been undertaken to examine the uncertainty of stock markets in Asia. As Franses and McAleer (2002) state, researchers are committedly seeking to model stock market volatility better, in order to forecast stock markets movements more accurately and possibly foresee such shocks. In light of prominent studies by Engle (1982), French et al. (1987), and Bollerslev (1987), the accumulated literature of financial econometrics indicates that, in addition to the set of economic variables suggested by Chen et al. (1986), stock market volatility has mainly been examined and estimated by time series volatility models.

Mandelbrot (1963) and Fama (1965) revealed that stock market volatility shows the volatility clustering property, a phenomenon which has been modeled by Engle’s (1982) ARCH model and its extension, Bollerslev’s (1986) GARCH model. For example, Bera and Higgins (1993) highlighted that the main contribution of the ARCH family models would be finding unconditional variance changes with time in the volatility of financial time series. On the other hand, Engle and Patton (2001) argued that “despite the success of GARCH models in capturing the salient features of conditional volatility, they have some undesirable characteristics” (p.244). The drawbacks of these models triggered the development of alternative specifications. As a result, options that consider asymmetric effects, such as EGARCH (Nelson 1991), PGARCH (Ding et al. 1993), and TGARCH (Zakoian 1994) have been introduced by researchers over the years. Furthermore, models that consider the long memory phenomenon have also been developed, such as FIGARCH (Baillie et al. 1996), FIEGARCH (Bollerslev and Mikkelsen 1996), CGARCH (Engle and Lee 1999), and HYGARCH (Davidson 2004). Although the success of the above models changes depending on the selected markets and time frames, it can be concluded that GARCH family models are powerful in estimating stock market volatility, confirming the studies of Chiang et al. (2000), Hung (2009), and Ahmed and Suliman (2011).

Some Asian markets have been deeply studied over the years using various models. Among these markets, Japan and China took the lead due to the rapid economic progress and explosive investments. Lux and Kaizoji (2007) studied the NIKKEI 225 Index from 1975 to 2001, and the findings showed that GARCH family models are able to present good forecast performance compared to naïve sample variance models, leading the authors to conclude that the time series models are well-suited for predicting large realizations of volatility. Ishida and Watanabe (2009) extended the research into the Japanese stock market by focusing minute-to-minute data with sample period spans from 1996 to 2007. They combined the GARCH model with ARFIMA and successfully predicted realized variance. On the other hand, Gu and Cen (2011) expanded the models for China’s stock market and the results revealed that GARCH and CGARCH models are preferred for more accurate prediction of volatility, while TGARCH and EGARCH are better to capture the asymmetric effects of the volatility behavior in China’s stock markets. They also suggested that GARCH-type models are more accurate and provide better forecasting compared to SV models for China’s capital markets. Meanwhile, Lin (2018) compared the adaptability of the GARCH models on the SSE Index and SX Index using daily returns from 2013 to 2017. Through empirical analysis and forecast evaluation, he discovered that the EGARCH model outperforms the ARCH, TARCH, GARCH and ARIMA models and it is more competent to predict volatility behavior in selected indices. For further research, see Guidi (2010), Chen and Wu (2011), Wei et al. (2018), Chaudhary et al. (2020), and Bhowmik and Wang (2020).

The ongoing argument over the performance of forecasting models has also leaped to emerging economies of Asia. The early findings about volatility behavior in ASEAN nations are fairly mixed. Wong and Kok (2005) compared the forecasting capabilities of six different models using daily returns from the ASEAN-5 equity markets (Indonesia, Malaysia, Singapore, Thailand and the Philippines) by covering the data from 2 January 1992 to 12 August 2002. They separated the results into pre-crisis, crisis and post-crisis periods. The findings suggest that the forecast results are most reliable for the pre-crisis and post-crisis periods and least reliable for the crisis period. Furthermore, the TARCH and ARCH-M models were found superior for the pre-crisis period, the ARCH-M and Random Walk models outperformed for crisis period, while the TARCH and EGARCH models were best for the post-crisis period for the selected ASEAN countries.

Likewise, Evans and McMillan (2007) examined the volatility forecasts of equity returns with a focus on asymmetric and long memory dynamics in more than 30 economies, including ASEAN-5 countries. The daily data for this study covered 11 years, from 1994 to 2005. By comparing 5 GARCH family and 4 simple pre-ARCH class of models, they found that the HYGARCH model performs best for Singapore, the CGARCH model for Thailand, and the EGARCH model for Indonesia, based on the RMSE error statistic. On the other hand, the moving average method provides the best forecast results for Malaysia and the exponential smoothing method is the best model for predicting the volatility of the Philippines stock market. Guidi and Gupta (2012) studied the same ASEAN-5 stock markets over the period from 2 January 2002 to 30 January 2012. They deployed the APARCH model under two different distributions to predict the volatility of the returns, and the empirical results revealed that the APARCH with the t-distribution is a good prediction model for the selected indices. They concluded that the Indonesian stock market has the largest response to volatility shocks among the ASEAN countries.

More recently, Anggita et al. (2020) investigated the stock market of Indonesia by using ARCH/GARCH models for the period of 2011–2017. The study concluded that the EGARCH model is superior compared to linear GARCH models in modelling and forecasting volatility in emerging markets. In a different study, Sharma et al. (2021) analyzed the top five emerging countries among the E7, including China and Indonesia, using linear and non-linear GARCH models for a period from 2000 to 2019 where the study results revealed that the GARCH model beat the non-linear GARCH models in all selected window periods, which supports the earlier findings of Srinivasan and Ibrahim (2010), but contradicts with Anggita et al. (2020). On the other hand, Lin (2018) showed the suitability of non-linear models for China’s stock market due to the significant properties of clustering and asymmetric events in the SSE Composite Index.

Although the reviewed literature has considerably enhanced our understanding of the forecasting performance of a variety of models and volatility behaviors in emerging and developed markets, the findings from the previous studies are quite unclear, given that they were highly dependent on the selection of countries and the range of data period. Thus, the current paper is expected to be one of the first empirical works regarding forecast comparison in ten Asian markets using three different time frames with 24 years of data, which includes two major crises that hit the selected economies at different magnitudes. Moreover, this research addresses the true nature of financial market volatility in countries that tend to be ignored, such as, the Philippines, Thailand, and Taiwan. In addition, identifying excess kurtosis by using student’s t-distribution and using recursive and rolling window methods for the selected GARCH models is expected to contribute to the gap in methodology in the field of stock market volatility of Asian countries.

## Methodology

### Empirical models

There are more than 300 GARCH-type models (Hansen and Lunde 2005) in the existing literature. Therefore, for brevity, the current paper is confined to focuses on the employed models only. In all selected models, the distributional assumption is considered under student’s t-distribution. The rationale behind this choice is that the asset returns are likely to follow levy distribution with fat tails, and the student’s t-distribution is more capable of accommodating fat tails compared to normal distribution, which reduces the potential considerable biases on the forecasting results (Andersen and Bollerslev 1998).

### GARCH model

The Generalized Autoregressive Conditional Heteroskedasticity (GARCH) model was developed and proposed by Tim Bollerslev in 1986. ARCH family models are a milestone in regression analysis in terms of estimating variance by a nonlinear estimation model. The GARCH model is based on a weighted average of past squared residuals with a few improvements compared to ARCH. First, GARCH has decaying weights on past squared residuals that stay above zero, no matter how much it falls. Second, it puts greater weight on more recent events. Third, it is superior for handling different sets of data in different frequencies. With these combined benefits, GARCH is an avant-garde model with a wide selection of extensions in predicting conditional volatility.

This model can be expressed with a mean specification and a variance specification. The GARCH (1,1) can be represented as follows:

Mean specification

1 $$r_{t} = \mu + \varepsilon_{t }$$

Variance specification

2 $$h_{t}^{2} = \alpha_{0} + \alpha_{1} \varepsilon_{t - 1}^{2} + \beta h_{t - 1}^{2}$$

where ${\alpha }_{0}>0$, ${\alpha }_{1}\ge 0$ and $\beta \ge 0$, and ${r}_{t}$ = asset return, $\mu$ = average return, ${\varepsilon }_{t}$= returns of residual.

Returns of residual can also be expressed as:

3 $${\varepsilon }_{t}={h}_{t}{z}_{t}$$

where ${z}_{t}$ is a random variable with zero mean and 1 variance (*i.i.d.*), and ${h}_{t}$ is the time-dependent standard deviation. For the GARCH (1,1) model, these two assumptions (${\alpha }_{1}\ge 0$, $\beta \ge 0$) are again needed to confirm that the conditional variance ${h}_{t}^{2}$ will have a non-negative value. To make sure that the model is covariance stationarity ${\alpha }_{1}+\beta <1$ is required.The mean specification is formed of by the aggregate of average term and error term. This process generates a one-period ahead estimate for the conditional variance ${h}_{t}^{2}$ which is a function of:

- Hypothetical long-run average variance: ${\alpha }_{0}$ (known as the constant term)
- First independent variable which reflects “news” about previous period volatility: ${\varepsilon }_{t-1}^{2}$ (known as ARCH term)
- Second independent variable which reflects forecast variance from previous period: ${\mathrm{h}}_{t-1}^{2}$ (known as GARCH term)

### GARCH-M model

Most models used in finance suppose that investors should be rewarded for taking additional risk by obtaining a higher return (Brooks 2008). Engle et al. (1987) proposed a new model to fit this theory, called GARCH in Mean (GARCH-M). This model is another variant of the GARCH-, class models with some extensions, which considers the conditional mean as a function of the conditional variance. The GARCH-M (1,1) model can be expressed by the two specifications as:

Mean specification

4 $${r}_{t}= \mu + \gamma {h}_{t}^{2}+{\varepsilon }_{t}$$

Variance specification

5 $${h}_{t}^{2}={a}_{0}+{a}_{1}{\varepsilon }_{t-1}^{2}+\beta {h}_{t-1}^{2}$$

The $\gamma$ parameter in the mean specification indicates risk premium coefficient. A positive $\gamma$ indicates that the conditional variance is positively correlated with the return and vice versa.

### EGARCH model

The Exponential GARCH model was proposed by Nelson (1991) based on the logarithmic version of conditional volatility. The benefit of the EGARCH model is that it places no restrictions on parameters, which allows negative coefficients in the model. Therefore, even if negative parameters exist in the equation, the conditional variance will remain positive. The EGARCH (1,1) equation is applied as follows:

6 $$\mathrm{ln}({h}_{t}^{2})= {a}_{0}+ {\beta }_{1}\mathrm{ln}\left({h}_{t-1}^{2}\right)+{a}_{1}\left\{\left|\frac{{\varepsilon }_{t-1}}{{h}_{t-1}}\right|-\sqrt{\frac{2}{\pi }}\right\}-\gamma \frac{{\varepsilon }_{t-1}}{{h}_{t-1}}$$

where the parameter $\gamma$ indicates the leverage effect which captures the impact of asymmetric news. If the leverage parameter $\gamma$ is positive, it demonstrates that good news (positive shock) will reduce the future volatility. However, when bad news (negative shock) increases future volatility, the leverage effect $\gamma$ will be negative and the term ${a}_{1}$ will capture the volatility clustering effect.

### TGARCH model

The Threshold GARCH model (also called as the GJR model) is one of the best-known and most commonly used asymmetric models to measure and handle with possible asymmetries, such as leverage effects. This model was developed by Zakoian (1994), but also studied by Glosten et al. (1993) as the Gloster-Jagannathan-Runkle GARCH (GJR-GARCH). In the TGARCH (1,1) model, the variance equation is defined as follows:

7 $${h}_{t}^{2}={a}_{0}+{a}_{1}{\varepsilon }_{t-1}^{2}+\gamma {D}_{t-1}{\varepsilon }_{t-1}^{2}+{\beta }_{1}{h}_{t-1}^{2}$$

where ${D}_{t-1}$ is a dummy variable to capture the leverage effect and

8 $${D}_{t-1}=\left\{\begin{array}{c}1 {\varepsilon }_{t-1}<0\quad bad\, news\\ 0 {\varepsilon }_{t-1}\ge 0\quad good\, news\end{array}\right.$$

where the term $$\gamma$$ is the leverage effect parameter. If $$\gamma =0$$, the specification above turns into the general GARCH (p, q) form. Apart from that, the impact of good news on volatility is $${a}_{1}$$, and the impact of bad news on volatility is $${a}_{1}+ \gamma$$. Thus, with a positive and significant leverage parameter ($$\gamma )$$, bad news has greater effect than good news on conditional volatility ($${h}_{t}^{2})$$.

### PGARCH model

The Power GARCH (PGARCH) model was developed by Ding, Granger, and Engle in 1993. The PGARCH model differentiates itself from the other asymmetric models by using conditional standard deviation instead of the conditional variance. The power parameter is defined as $\theta$ and ${h}_{t}^{\theta }$ is used instead of ${h}_{t}^{2}$. The model is defined as follows:

9 $${h}_{t}^{\theta }={a}_{0}+\sum_{k=1}^{p}{\beta }_{k}{h}_{t-1}^{\theta }+\sum_{l=1}^{q}{{a}_{l}\left(\left|{\varepsilon }_{t-l}\right|-{\gamma }_{l}{\varepsilon }_{t-1}\right)}^{\theta }$$

where ${a}_{l}$ = standard ARCH parameter, ${\beta }_{k}$ = standard GARCH parameter, ${\gamma }_{l}$ = leverage parameter.

The leverage parameter ${\gamma }_{l}$ captures the asymmetric effects of previous shocks. When the power parameter $\theta =2$, the equation turns into a classic GARCH model, and when $\theta =1$, the model estimates conditional standard deviation instead of conditional variance.

### Forecasting method

Out-of-sample tests are widely considered as the “gold standard” of the forecast evaluation, and according to the “conventional wisdom”, the forecasts of the estimated models should be evaluated by conducting out-of-sample fit rather than generating the same set of data that was used to estimate the model’s parameters, which is called an “in-sample” forecast. Bartolomei and Sweet (1989) and Pant and Starbuck (1990) show that even the best in-sample forecasts may not be successful to forecast post-sample data. Furthermore, throughout the empirical studies, in-sample forecasting performance is found to be less reliable compared to out-of-sample tests, which may be due to the vulnerability to outliers and data mining (White, 2000). Therefore, out-of-sample forecast is seen as the “ultimate test of forecasting model” by econometricians and forecasters (Stock and Watson 2015, p. 571).

Out-of-sample forecasts can be estimated using two different methods which are known as recursive forecast and rolling window forecast. The recursive forecast sets a fixed initial sample data starting from $t=1,\dots ,T$ to fit the models, and $L$ step ahead forecast is computed for out-of-sample prediction starting from time $T$ until no more $L$ step ahead forecast can be counted. On the other hand, the rolling window forecast sets fixed initial sample data starting from $t=1,\dots ,T$ to estimate the model and specify the window length. Out-of-sample forecast begins from time $T$, and both the start and the end estimation dates consecutively increase by one observation where the model is re-estimated each time from $t=2,\dots ,T+1$. $L$ step ahead out-of-sample forecast is computed beginning with time $T+1$ until no more $L$ step ahead forecast can be counted.

For each index, forecasting models are estimated using recursive and rolling window methods and assessed by out-of-sample performance. The maximum likelihood method has been used to estimate parameters. The choice of window size for out-of-sample forecasting is controversial, since there is no satisfactory solution for the optimal length. However, to keep the competence of the estimated parameters robust and avoid non-convergence problems, adequately large estimation size is recommended, especially in the applications of richly parameterized GARCH family models (Peseran and Timmerman 2007; and Inoue et al. 2014). Therefore, the whole sample period is divided into two samples in each frequency and a hold-out sample for the out-of-sample forecast is chosen as a second half, with parameters estimated based on the first half. In this context, a similar procedure has been followed with earlier works, such as those of Akgiray (1989), Pagan and Schewert (1990), Brailsford and Faff (1996), and Brooks (1998). Sample periods and sample sizes can be seen in Table 1.

**Table 1:** Sample periods and sample sizes for selected countries and frequencies

Country	Frequency	Estimation period	Estimation size	Forecast period	Forecast size	Full sample size
Japan	Daily	12/09/1994 8/11/2006	2874	8/14/2006 5/02/2018	2876	5750
	Weekly	9/23/1994 7/14/2006	616	7/21/2006 4/27/2018	617	1233
	Monthly	1993m10 2006m02	147	2006m03 2018m04	149	296
Singapore	Daily	8/31/1999 12/30/2008	2344	12/31/2008 5/02/2018	2346	4690
	Weekly	9/03/1999 1/09/2009	486	1/16/2009 4/27/2018	489	975
	Monthly	1999m08 2009m01	112	2009m02 2018m04	114	226
Hong Kong	Daily	1/10/1995 8/29/2006	2874	8/30/2006 5/03/2018	2877	5751
	Weekly	10/21/1994 8/04/2006	614	8/11/2006 5/04/2018	616	1230
	Monthly	1993m10 2006m02	147	2006m03 2018m04	149	296
Malaysia	Daily	1/10/1995 9/04/2006	2871	9/05/2006 4/30/2018	2869	5740
	Weekly	10/21/1994 8/04/2006	612	8/11/2006 4/20/2018	616	1228
	Monthly	1993m10 2006m02	147	2006m03 2018m04	149	296
Indonesia	Daily	1/11/1995 8/25/2006	2847	8/28/2006 4/26/2018	2841	5688
	Weekly	10/21/1994 7/28/2006	603	8/04/2006 2/09/2018	615	1218
	Monthly	1993m10 2006m02	147	2006m03 2018m04	149	296
Thailand	Daily	1/11/1995 8/24/2006	2855	8/25/2006 4/25/2018	2848	5703
	Weekly	10/21/1994 8/04/2006	613	8/11/2006 4/27/2018	616	1229
	Monthly	1993m10 2006m02	147	2006m03 2018m04	149	296
China	Daily	1/10/1995 9/11/2006	2828	9/12/2006 5/03/2018	2829	5657
	Weekly	10/21/1994 9/29/2006	593	10/13/2006 5/04/2018	596	1189
	Monthly	1993m10 2006m02	147	2006m03 2018m04	149	296
Taiwan	Daily	1/11/1995 3/23/2006	2997	3/24/2006 5/02/2018	2895	5892
	Weekly	10/22/1994 7/22/2006	606	7/29/2006 4/28/2018	608	1214
	Monthly	1993m10 2006m02	147	2006m03 2018m04	149	296
South Korea	Daily	1/10/1995 5/02/2006	2970	5/03/2006 5/02/2018	2973	5943
	Weekly	10/23/1994 7/30/2006	613	8/06/2006 4/29/2018	615	1228
	Monthly	1993m10 2006m02	147	2006m03 2018m04	149	296
Philippines	Daily	1/11/1995 7/17/2006	2875	7/18/2006 5/02/2018	2853	5728
	Weekly	10/28/1994 8/04/2006	612	8/11/2006 5/04/2018	616	1228
	Monthly	1993m10 2006m02	147	2006m03 2018m04	149	296

The number of observations in full sample size varies due to the differences in trading days per year depending on the country

### Forecast performance evaluation

Great decisions are based on great forecasts. There are a wide selection of procedures available in the literature to evaluate the most accurate forecasts. In this study, the most common and important error measures are chosen to evaluate the predictive accuracy of selected volatility models. Nevertheless, there is no consensus about which error function is more suitable to assess the models. Therefore, instead of focusing on a single criteria, five different loss functions were determined for producing forecasts. These loss functions are Mean Absolute Error (MAE), Mean Absolute Percentage Error (MAPE), Root Mean Square Error (RMSE), Quasi-Likelihood (QLIKE) and Mean Squared Error (MSE).

### Mean absolute error (MAE)

MAE measures the average magnitude of the errors in a set of predictions, without considering their direction. It is the average over the test sample of the absolute differences between prediction and actual observation where all individual differences have equal weight. The mean absolute error is given by:

10 $$\mathrm{MAE}=\frac{1}{n}\sum_{t=1}^{n}\left|{\sigma }_{t}^{2}-{\widehat{\sigma }}_{t}^{2}\right|$$

where *n* denotes the rank of forecasted data, ${\sigma }_{t}^{2}$ is the true volatility series which is obtained by the squared return series and ${\widehat{\sigma }}_{t}^{2}$ is the forecasted conditional variance at time $t$ acquired by using GARCH family models.

### Mean absolute percentage error (MAPE)

MAPE is the sum of the individual absolute errors divided by each period separately. In other words, it is the average of the percentage errors. The advantage of the MAPE is that it is easy to interpret and helpful for comparing the performance of the estimated volatility models. The mean absolute percentage error is defined as follows:

11 $$\mathrm{MAPE}=\frac{1}{n}\sum_{t=1}^{n}\frac{\left|{\sigma }_{t}^{2}-{\widehat{\sigma }}_{t}^{2}\right|}{{\sigma }_{t}^{2}}$$

### Root mean square error (RMSE)

RMSE is the square root of the average of squared differences between prediction and actual observation. Since the errors are squared before they are averaged, the RMSE gives a relatively high weight to large errors. This means the RMSE is most useful when large errors are particularly undesirable. Its value can only be positive, and a value of zero (almost never achieved in practice) would indicate a perfect fit to the data. In general, a lower RMSE is better than a higher one. However, comparisons across different types of data would be invalid because the measure is dependent on the scale of the numbers used. The following formula is given for the root mean square error:

12 $$\mathrm{RMSE}=\sqrt{\frac{1}{n}\sum_{t=1}^{n}{\left({\sigma }_{t}^{2}-{\widehat{\sigma }}_{t}^{2}\right)}^{2}}$$

### Quasi-likelihood loss function (QLIKE)

The term quasi-likelihood function was introduced by Wedderburn (1974) to describe a function that has similar properties to the log-likelihood function. In QLIKE loss function, the mean and the variance is specified in the form of a variance function giving the variance as a function of the mean.

13 $$\mathrm{QLIKE}=\frac{1}{n}\sum_{t=1}^{n}\left(\mathrm{log}\left({\widehat{\sigma }}_{t}^{2}\right)+\left(\frac{{\sigma }_{t}^{2}}{{\widehat{\sigma }}_{t}^{2}}\right)\right)$$

Patton and Sheppard (2009), Patton (2011), and Conrad and Kleen (2018) revealed that the squared error loss tends to be more sensitive to extreme observations than QLIKE, which provides further motivation for using QLIKE in volatility forecasting applications.

### Mean squared error (MSE)

MSE is another popular accuracy measure in the empirical financial literature developed by Bollerslev et al. (1994) to gauge the forecasting performance of volatility models. As a distinctive feature, it has the tendency of penalizing large forecast errors compared to other loss functions, and thus it is recognized as one of the most appropriate measures in terms of dealing with imperfect volatility proxy (Patton 2011). The mean squared error is given as follows:

14 $$\mathrm{MSE}=\frac{1}{n}\sum_{t=1}^{n}{\left({\sigma }_{t}^{2}-{\widehat{\sigma }}_{t}^{2}\right)}^{2}$$

### Forecast comparison test (DM-test)

In order to evaluate the predictive accuracy of two competing models, the Diebold-Mariano test (hereafter, the DM test) is employed. Diebold and Mariano (2002) introduced an approach for testing of the null hypothesis of no difference for the equal forecast accuracies between two sets of competing models. The test can be applied with any error criterion such as straight differences, absolute differences or squared differences. Furthermore, it is able to incorporate autocorrelation between the given series. The DM test is widely employed in the empirical finance literature with various adaptations: see Xekalaki and Stavros (2010), Curto and Pinto (2012), Gilleland and Roux (2015), and Coroneo and Iacone (2018).

Consider two sets of competing forecast sequences, defined as:

15 $$\left\{{f}_{it}:t=1, 2,\dots T\right\}, i=1, 2$$

and define the equation of difference between actual value ${y}_{t}$ $\left\{{y}_{t}:t=1, 2,\dots T\right\}$ and the predicted value ${f}_{it}$ as

16 $${e}_{it}={f}_{it}-{y}_{t}$$

The accuracy of each forecast is gauged by the loss function:

17 $$L\left({e}_{it}\right)={e}_{it}^{2}$$

The loss functions adopted for this study are the absolute-error loss function:

18 $${L}_{1}\left({e}_{it}\right)=\sum_{t=1}^{T}\left|{e}_{it}\right|$$

and the Squared-error loss function:

19 $${L}_{2}\left({e}_{it}\right)=\sum_{t=1}^{T}{{(e}_{it})}^{2}$$

and the loss differential between the two forecasts is defined by:

20 $${d}_{t}=L\left({e}_{1t}\right)-L\left({e}_{2t}\right)$$

To assess whether the two competing forecasts have same predictive ability, the equal accuracy hypothesis is considered. The null hypothesis of DM test is given as:

21 $${H}_{0}:E\left({d}_{t}\right)=0$$

versus the two-sided alternative hypothesis of one of the two forecasts have better accuracy:

22 $${H}_{1}:E\left({d}_{t}\right)\ne 0$$

**Fig. 1:**
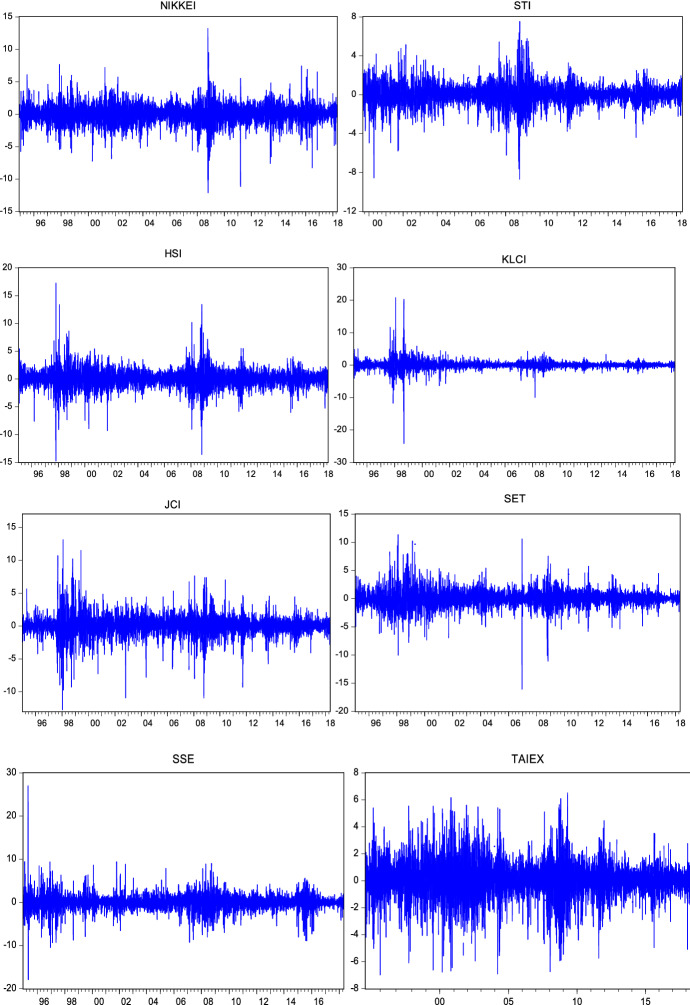

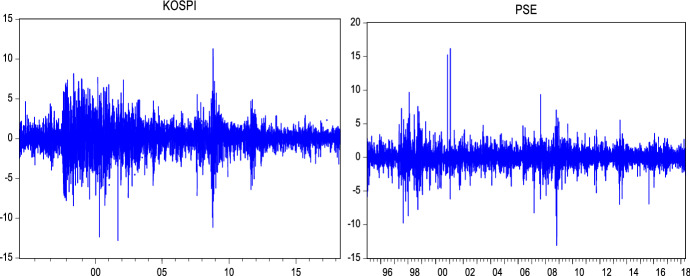
Daily log returns

Then, the DM test statistic can be expressed as:

23 $$DM=\frac{\overline{d}}{\sqrt{\widehat{\omega }/T}}\sim N\left(\mathrm{0,1}\right)$$

where $\overline{d }=\frac{1}{T}\sum_{t=1}^{T}{d}_{t}=\frac{1}{T}\sum_{t=1}^{T}\left[L\left({e}_{1t}\right)-L\left({e}_{2t}\right)\right]$ and $\widehat{\omega }$ is a consistent estimator of the asymptotic variance of $\overline{d}\sqrt{T }$. The null hypothesis of ${H}_{0}$ is rejected if $\left|DM\right|>1.96$ which can be shown in Fig. 1 as area A and area C. Conversely, the null hypothesis of ${H}_{0}$ cannot be rejected in the event of $\left|DM\right|\le 1.96$.

## Data

Asia is divided into two regions: developed and emerging economies. The highly developed countries include Japan and the four Asian Tigers—Hong Kong, South Korea, Taiwan, and Singapore. China and Malaysia are other major economic forces which are considered an important powerhouse in the region, however, academics often classify these countries as “developing”; see Johansson and Ljungwall (2009), Luo et al. (2010), Jayasuriya (2011), Zhang et al. (2013), and Li and Giles (2015). Besides this, the Shanghai Stock Exchange was founded in 1990, 99 years after the Hong Kong Stock Exchange, which was founded in 1891. Even today, most of mainland Chinese companies are listed in Hong Kong. Therefore, the Chinese stock market will be evaluated in the emerging markets category.

In this study, ten Asian countries have been selected for investigation and their widely accepted indices have been chosen. The five developed market indices that have been added are as follows: the Nikkei 225 Index (NIKKEI) from Japan, the Hang Seng Index (HSI) from Hong Kong, the Korea Composite Stock Market Index (KOSPI) from South Korea, the Taiwan Capitalization Weighted Stock Index (TAIEX) from Taiwan, and the Straits Times Index (STI) from Singapore. The remaining five Asian countries are chosen as emerging markets and their broadly accepted stock market indices are considered as follows: the SSE Composite Index (SSE) from China, the PSE Composite Index (PSE) from the Philippines, the Stock Exchange of Thailand Index (SET) from Thailand, the Kuala Lumpur Composite Index (KLCI) from Malaysia, and the Jakarta Stock Exchange Composite Index (JCI) from Indonesia.

Daily, weekly and monthly time series data is obtained from the Bloomberg database to ensure the reliability and accuracy of older data. The overall sample period covers 25 years in total, starting from November 1993 to May 2018. However, one problem was the limitation on accessing older data in higher time frames, and thus the daily and weekly data start from 1994 instead of 1993. Another challenge was non-synchronous holidays in different markets which may cause computational difficulties and negatively effect the output of the models. Therefore, the data range has been chosen separately for each market to not get exposed to data loss. The statistical software Eviews 10 was used for the quantitative analysis.

The main advantage of daily data is providing more information in terms of estimating volatility for applied econometric models since they are more data-intensive than simple regression models. Weekly and monthly frequencies are also estimated, since they provide a broader framework regarding volatility, and it is crucial to understand the comparison between different frequencies. In order to satisfy stationarity, closing price series are transformed to return series in all daily, weekly and monthly time frames for each index.

**Fig. 2:**
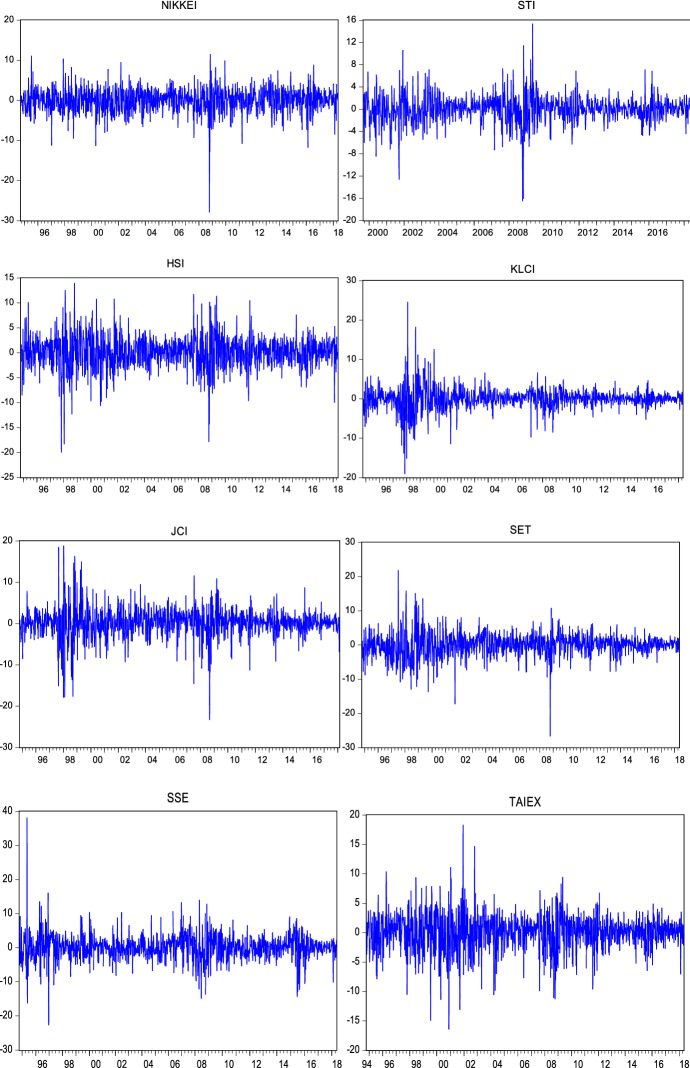

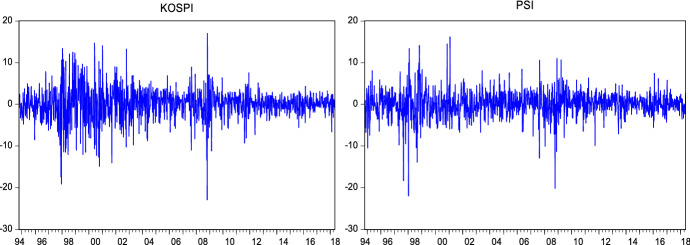
Weekly log returns

**Fig. 3:**
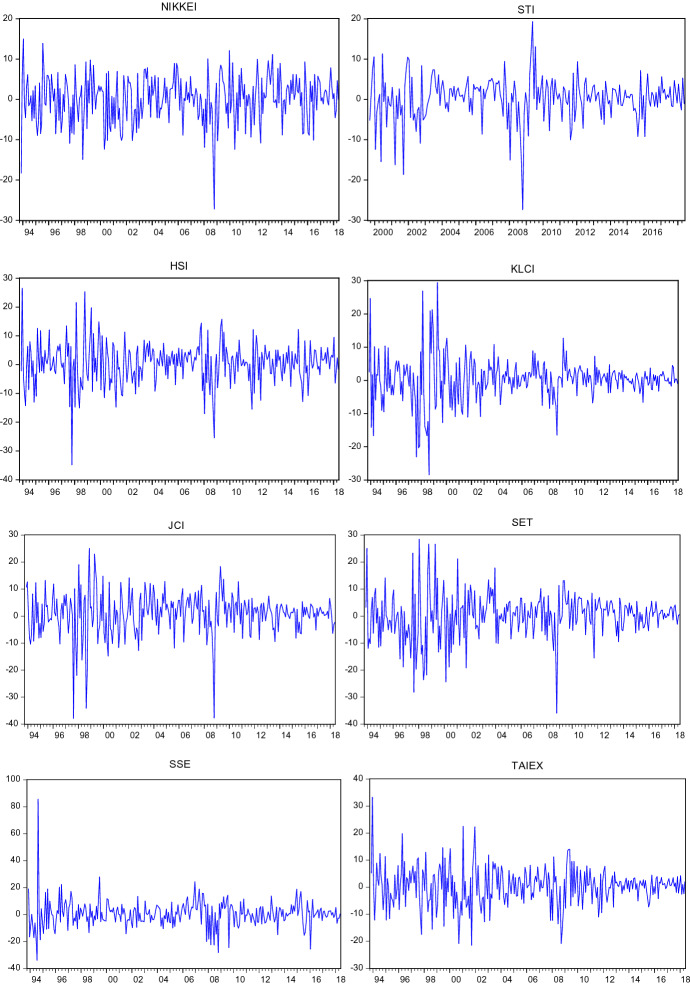

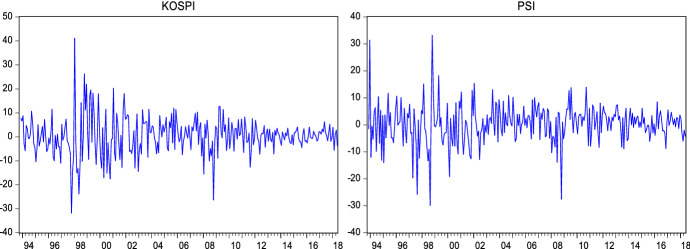
Monthly log returns

Return series have been obtained as shown in the following formula:

24 $${R}_{t}=log({P}_{t}/{P}_{t-1})*100$$

where ${R}_{t}$ denotes the logarithmic return at time $t$. ${P}_{t}$ and ${P}_{t-1}$ are the closing price of the index at time $t$ and $t-1$ respectively. Figures 1, 2 and 3 show that the return series are fluctuating around zero, which is evidence of the volatility clustering phenomenon.

Table 2 reports the descriptive statistics of the in-sample period for each frequency. According to the tables, the mean and median are centered around zero in the daily return series, while with the reducing frequency the tendency of deviation increases, which is expected. Looking at the skewness of the series, the NIKKEI and STI indices have negative values for all selected time frames which implies asymmetric distributions skewed to the left, while the KLCI, SET and SSE indices report positive skewness for each frequency suggesting asymmetric distributions skewed to the right. For the remaining five indices, the direction of skewness changes depending on the selected frequency. Where the kurtosis is concerned, the given values from all tables indicate a leptokurtic characteristic, which signifies the existence of fatter tails. Lastly, the Jarque–Bera test statistic for normality rejects the null hypothesis that returns follow a normal distribution.

**Table 2:** Summary of in-sample descriptive statistics for daily, weekly and monthly return series

	Nikkei	Straits Times Index	Hang Seng Index	Kuala Lumpur Composite Index	Jakarta Composite Index	Set Index	SSE Index	Taiex	Kospi	Pse Index
Mean	− 0.006896	− 0.008975	0.02846	0.001518	0.039814	− 0.02413	0.035713	0.013712	0.012371	− 0.011758
Median	− 0.002262	0.028592	0.036591	0.002292	0.058451	− 0.058689	0.025033	− 0.00769	0.050867	− 0.038199
Maximum	7.660481	7.531083	17.2471	20.81737	13.12768	11.34953	26.99277	6.172055	8.16129	16.1776
Minimum	− 7.233984	− 8.695982	− 14.73468	− 24.15339	− 12.73214	− 10.02803	− 17.90509	− 6.975741	− 12.8047	− 9.744158
Std. Dev	1.440705	1.323858	1.641641	1.632034	1.695683	1.753001	1.817131	1.53731	1.990682	1.503186
Skewness	-0.022372	− 0.398517	0.119978	0.594985	0.032216	0.497725	0.876601	− 0.055486	− 0.189095	0.755579
Kurtosis	4.863499	7.481252	14.40016	46.90451	10.89784	7.216725	27.64449	4.889879	5.932565	15.04423
Jarque–Bera	416.2318	2024.212	15,564.62	230,598.5	7384.241	2227.573	71,902.92	432.3149	1082.671	17,515.88
Probability	0.0000	0.0000	0.0000	0.0000	0.0000	0.0000	0.0000	0.0000	0.0000	0.0000
Observations	2875	2345	2873	2869	2841	2848	2827	2895	2972	2853
Sample	12/09/1994 8/11/2006	8/31/1999 12/30/2008	1/10/1995 8/29/2006	1/10/1995 9/04/2006	1/11/1995 8/25/2006	1/11/1995 8/24/2006	1/10/1995 9/11/2006	1/11/1995 3/23/2006	1/10/1995 5/02/2006	1/11/1995 7/17/2006

	Nikkei	Straits Times Index	Hang Seng Index	Kuala Lumpur Composite Index	Jakarta Composite Index	SET Index	SSE Index	Taiex	Kospi	PSE Index
Mean	− 0.04703	− 0.036714	0.096331	− 0.027284	0.16798	− 0.125584	0.151207	− 0.008768	0.028492	− 0.04337
Median	0.070721	0.10386	0.196187	0.023564	0.22806	− 0.009112	0.1595	0.202186	0.197534	0.044041
Maximum	11.04704	11.43806	13.9169	24.57857	18.80297	21.83839	38.07101	18.31817	14.70595	16.18463
Minimum	− 11.29215	− 16.46836	− 19.92123	− 19.02678	− 17.8541	− 17.24383	− 22.6293	− 16.40812	− 19.14189	− 21.98549
Std. Dev	2.874453	2.967102	3.499748	3.428855	4.091328	4.134383	3.918802	3.590463	4.507016	3.587097
Skewness	− 0.081482	− 0.724046	− 0.471968	0.278927	− 0.049843	0.219675	1.295237	− 0.150573	− 0.199036	− 0.264552
Kurtosis	3.901964	7.396589	6.420523	11.41934	6.898372	5.431559	19.9507	5.59161	4.444065	7.697924
Jarque–Bera	21.56245	435.6816	322.6443	1824.41	389.0519	156.4536	7289.664	172.1637	57.40346	572.7302
Probability	0.0000	0.0000	0.0000	0.0000	0.0000	0.0000	0.0000	0.0000	0.0000	0.0000
Observations	616	488	615	615	614	615	595	607	614	615
Sample	9/23/1994 7/14/2006	9/03/1999 1/09/2009	10/21/1994 8/04/2006	10/21/1994 8/04/2006	10/21/1994 7/28/2006	10/21/1994 8/04/2006	10/21/1994 9/29/2006	10/22/1994 7/22/2006	10/23/1994 7/30/2006	10/28/1994 8/04/2006

	Nikkei	Straits Times Index	Hang Seng Index	Kuala Lumpur Composite Index	Jakarta Composite Index	SET Index	SSE Index	Taiex	Kospi	PSE Index
Mean	− 0.132043	− 0.198429	0.361043	− 0.030609	0.655951	− 0.360376	0.315205	0.312537	0.407225	− 0.084081
Median	0.582549	0.901965	0.94774	0.192732	0.609101	− 0.200538	-0.10265	0.337473	− 0.174881	− 0.150691
Maximum	14.96626	11.31864	26.45214	29.44212	25.01933	28.42753	85.52026	33.23789	41.0616	33.16657
Minimum	− 18.30893	− 27.36404	− 34.82366	-28.4632	-37.8555	− 28.16608	− 34.03195	− 21.50303	− 31.81042	− 29.89063
Std. Dev	5.856204	6.214737	8.024243	8.577219	9.078728	10.06465	11.0501	8.325613	9.702619	8.49973
Skewness	− 0.259136	− 1.347471	− 0.132261	0.145642	− 0.780137	0.122933	3.041904	0.371143	0.38633	0.210962
Kurtosis	2.867726	6.212182	5.671145	5.047541	5.767228	3.794966	25.90612	4.319366	4.991361	5.865271
Jarque–Bera	1.764292	82.77637	44.43075	26.37649	62.23404	4.269925	3463.836	14.13225	28.13555	51.72477
Probability	0.4139	0.0000	0.0000	0.0000	0.0000	0.1182	0.0000	0.0009	0.0000	0.0000
Observations	148	113	148	148	148	148	148	148	148	148
Sample	1993m10 2006m02	1999m08 2009m01	1993m10 2006m02	1993m10 2006m02	1993m10 2006m02	1993m10 2006m02	1993m10 2006m02	1993m10 2006m02	1993m10 2006m02	1993m10 2006m02

Mean, Median, Maximum, Minimum and Standard Deviation are multiplied by 100 (% format)

## Empirical results

Table 3 demonstrates the forecasting performance for daily return series based on the calculation of Mean Absolute Error (MAE), Mean Absolute Percentage Error (MAPE), Root Mean Squared Error (RMSE), Quasi-Likelihood (QLIKE) and Mean Squared Error (MSE) using the recursive approach. The overall results of forecasting performance inform that the EGARCH and TGARCH models perform better than the rest of the models in the HANG SENG, STI, SET, JCI, TAIEX, KOSPI and PSE indices. These findings are also in line with the studies of Liu and Morley (2009), and Wei-Chong et al. (2011), in which they find that asymmetric models outperform in the stock markets of Hong Kong and Japan respectively. The results also indicate that the GARCH-M model outperforms in KLCI index, based on MAE and QLIKE statistics, while the GARCH and GARCH-M models equally outperform in the SSE index, which is steady with the findings of Liu et al. (2009). The KLCI index is the only index that shows mixed results, since EGARCH has minimum values for both RMSE and MSE loss functions while GARCH-M indicates the smallest numbers under the MAE and QLIKE statistics. Lim and Sek (2013) had similar results on the Malaysian stock market, which shows that the Malaysian market tends to produce more complicated results and requires more detailed examination.

**Table 3:** Comparison of recursive forecast performance measures for daily return series

	MAE	MAPE	RMSE	QLIKE	MSE
Nikkei Index					
GARCH (1,1)	2.587036	104.8954	6.927287	1.562947	0.4798731
EGARCH (1,1)	**2.479166**	105.4572	6.880609	**1.536782**	0.4734279
TGARCH (1,1)	2.580439	**103.9552**	**6.801676**	1.542612	**0.4626279**
GARCH-M (1,1)	2.586122	104.9069	6.927472	1.562966	0.4798987
PGARCH (1,1)	2.527196	105.0626	6.869496	1.541884	0.4718997
Hang Seng Index					
GARCH (1,1)	2.561509	103.1643	7.264017	1.462073	0.5276595
EGARCH (1,1)	**2.484555**	102.2727	**6.958204**	1.456995	**0.4841661**
TGARCH (1,1)	2.528717	**101.3025**	6.991431	**1.449444**	0.4888010
GARCH-M (1,1)	2.559787	103.1133	7.251374	1.461458	0.5258242
PGARCH (1,1)	2.586652	101.3267	6.995891	1.462087	0.4894248
Straits Times Index					
GARCH (1,1)	**0.913886**	96.78284	1.976149	0.496195	0.0390516
EGARCH (1,1)	0.910986	**95.03708**	**1.972934**	**0.491629**	**0.0389246**
TGARCH (1,1)	0.924262	95.70385	1.980209	0.495537	0.0392122
GARCH-M (1,1)	0.914155	96.81738	1.976955	0.496303	0.0390835
PGARCH (1,1)	0.951394	95.25337	2.01328	0.497664	0.0405329
Set Index					
GARCH (1,1)	1.906175	105.3177	6.7179	1.206068	0.4513018
EGARCH (1,1)	**1.853941**	105.0882	**6.676276**	**1.153943**	**0.4457266**
TGARCH (1,1)	1.894362	**103.8586**	6.676794	1.181998	0.4457958
GARCH-M (1,1)	1.88913	105.4373	6.712436	1.20002	0.4505679
PGARCH (1,1)	1.951078	104.5026	6.756292	1.170807	0.4564748
Kuala Lumpur Composite Index					
GARCH (1,1)	0.679296	99.98197	2.277337	0.060023	0.0518626
EGARCH (1,1)	0.667403	100.4669	**2.235911**	0.053187	**0.0499929**
TGARCH (1,1)	0.701806	**99.28575**	2.313005	0.062458	0.0534999
GARCH-M (1,1)	**0.65096**	100.0444	2.254335	**0.044328**	0.0508202
PGARCH (1,1)	0.69291	99.79142	2.276221	0.062842	0.0518118
Jakarta Composite Index					
GARCH (1,1)	2.0441	101.4464	5.259287	1.197184	0.2766010
EGARCH (1,1)	**1.996598**	100.9008	**5.176766**	**1.184489**	**0.2679890**
TGARCH (1,1)	2.037848	**99.93267**	5.222167	1.193907	0.2727103
GARCH-M (1,1)	2.043408	101.4626	5.256121	1.196861	0.2762681
PGARCH (1,1)	2.041875	100.2851	5.226898	1.195188	0.2732046
SSE Composite Index					
GARCH (1,1)	3.362493	**104.7333**	6.933591	1.697384	0.4807468
EGARCH (1,1)	3.337777	105.1576	**6.908015**	1.686254	**0.4772067**
TGARCH (1,1)	3.369595	104.872	6.924792	1.690698	0.4795275
GARCH-M (1,1)	**3.331011**	104.787	6.914063	1.687951	0.4780427
PGARCH (1,1)	3.384766	104.7707	6.951167	**1.683974**	0.4831872
Taiex Index					
GARCH (1,1)	1.565298	99.75422	3.162739	1.028196	0.1000292
EGARCH (1,1)	**1.55803**	98.361	**3.141356**	**1.018578**	**0.0986812**
TGARCH (1,1)	1.600145	**96.6748**	3.159284	1.024245	0.0998107
GARCH-M (1,1)	1.565305	99.73742	3.161918	1.028516	0.9997723
PGARCH (1,1)	1.617696	97.8082	3.197254	1.029186	0.1022244
Kospi Index					
GARCH (1,1)	1.790022	100.6294	4.913339	1.005362	0.241409
EGARCH (1,1)	**1.767842**	100.9281	**4.821477**	0.996488	**0.2324664**
TGARCH (1,1)	1.792983	**98.68959**	4.854616	**0.993382**	0.235673
GARCH-M (1,1)	1.789475	100.7094	4.912992	1.005631	0.2413749
PGARCH (1,1)	1.83218	99.27255	4.895882	1.003648	0.2396966
PSE Composite Index					
GARCH (1,1)	1.844115	98.15845	5.102734	1.237161	0.260379
EGARCH (1,1)	**1.808021**	97.34699	**5.021987**	**1.222524**	**0.2522036**
TGARCH (1,1)	1.858834	97.14607	5.08543	1.226648	0.258616
GARCH-M (1,1)	1.853516	98.17373	5.101353	1.241222	0.260238
PGARCH (1,1)	1.847766	**97.04785**	5.05302	1.223938	0.2553301

The table presents results from estimating regressions of volatility for each model and market. Columns indicate the particular loss functions, while the rows show corresponding volatility models under the selected markets. Numbers in bold demonstrate the minimum forecast error (preferred model)

On the other hand, based on Table 4 which reports the results for the rolling window method, there is no symmetric model that performs better than the asymmetric models. Asymmetric models dominate in all the selected markets, with the leading of EGARCH model except for the HANG SENG index, where the PGARCH model has clear superiority based on the four out of five statistics. The reason may arise from these two issues. First, due to the nature of symmetric GARCH models, they are not able to capture the leverage effect of volatility, and Asian stock markets tend to exhibit volatility asymmetry phenomenon. Second, the rolling window method does not allow the use of all available data to generate forecasts as in the recursive method, which may lead to potential estimation problems. However, as Table 3 shows, asymmetric models have superiority in most of the indices as well. The values between recursive and rolling window methods are highly mixed. Regardless of the models, a comparison cannot be conducted based on the error statistics since each method provides results in its own terms. Therefore, daily results do not suggest any significant superiority between these two methods.

**Table 4:** Comparison of rolling window forecast performance measures for daily return series

	MAE	MAPE	RMSE	QLIKE	MSE
Nikkei Index					
GARCH (1,1)	2.604888	106.2082	6.948597	1.562196	0.48283
EGARCH (1,1)	**2.475121**	**105.0707**	6.861767	**1.523355**	0.4708385
TGARCH (1,1)	2.592102	105.2052	**6.824228**	1.545574	**0.4657008**
GARCH-M (1,1)	2.604134	106.2296	6.949402	1.562222	0.4829418
PGARCH (1,1)	2.554392	105.7776	6.887053	1.545749	0.474315
Hang Seng Index					
GARCH (1,1)	2.50233	104.4677	7.25271	1.459778	0.526018
EGARCH (1,1)	**2.415606**	104.2941	7.049965	1.447725	0.49702
TGARCH (1,1)	2.455935	104.1706	7.008374	1.445925	0.491173
GARCH-M (1,1)	2.500407	104.3844	7.23523	1.458975	0.5234855
PGARCH (1,1)	2.432685	**104.1031**	**6.97589**	**1.443245**	**0.4866304**
Straits Times Index					
GARCH (1,1)	0.902532	98.64766	1.972595	0.493296	0.0389113
EGARCH (1,1)	**0.898874**	97.70559	1.975188	0.487509	0.0390136
TGARCH (1,1)	0.902942	96.84493	**1.970554**	**0.481714**	**0.0388308**
GARCH-M (1,1)	0.902158	98.66443	1.973111	0.493004	0.0389316
PGARCH (1,1)	0.928567	**97.36197**	1.997833	0.494192	0.0399133
Set Index					
GARCH (1,1)	1.882887	105.6482	6.710712	1.201288	0.4503365
EGARCH (1,1)	**1.840981**	104.8121	6.707536	1.161184	0.4499104
TGARCH (1,1)	1.885422	**103.8788**	**6.67502**	1.180261	**0.4455589**
GARCH-M (1,1)	1.874154	105.8696	6.711776	1.199131	0.4504793
PGARCH (1,1)	1.900219	104.6202	6.74088	**1.158713**	0.4543946
Kuala Lumpur Composite Index					
GARCH (1,1)	0.651199	101.0546	2.265128	0.038902	0.0513080
EGARCH (1,1)	**0.646715**	**99.94341**	**2.232009**	**0.033492**	**0.0498186**
TGARCH (1,1)	0.669354	100.6234	2.302587	0.038076	0.0530190
GARCH-M (1,1)	0.646861	101.3661	2.260683	0.038132	0.0511068
PGARCH (1,1)	0.652764	100.3483	2.242972	0.039637	0.0503092
Jakarta Composite Index					
GARCH (1,1)	2.026501	101.0445	5.243836	1.194157	0.2749781
EGARCH (1,1)	**1.989233**	99.66616	**5.164053**	**1.186056**	**0.2666745**
TGARCH (1,1)	2.0316	**98.96727**	5.207676	1.195521	0.2711989
GARCH-M (1,1)	2.029872	101.0232	5.248425	1.19493	0.2754597
PGARCH (1,1)	2.016035	99.50494	5.193848	1.191246	0.2697605
SSE Composite Index					
GARCH (1,1)	3.193871	109.9976	6.867032	1.677965	0.4715613
EGARCH (1,1)	**3.169549**	**109.723**	**6.859287**	**1.673801**	**0.4704982**
TGARCH (1,1)	3.192872	109.9885	6.867631	1.677937	0.4716435
GARCH-M (1,1)	3.194051	109.9939	6.866919	1.677952	0.4715458
PGARCH (1,1)	3.197683	109.8056	6.873206	1.674948	0.4724097
Taiex Index					
GARCH (1,1)	1.544914	102.0293	3.16788	1.019933	0.1003546
EGARCH (1,1)	**1.535502**	100.4965	**3.157702**	**1.004684**	**0.0997108**
TGARCH (1,1)	1.582406	**98.35006**	3.163942	1.011804	0.1001053
GARCH-M (1,1)	1.544532	102.0356	3.167174	1.019876	0.1003099
PGARCH (1,1)	1.586746	99.80402	3.190685	1.015073	0.1018047
Kospi Index					
GARCH (1,1)	1.793941	100.3222	4.914529	1.00288	0.241526
EGARCH (1,1)	**1.785204**	97.88502	**4.809564**	**0.998141**	**0.231319**
TGARCH (1,1)	1.816324	96.6203	4.861871	0.998325	0.2363779
GARCH-M (1,1)	1.794187	100.4138	4.918188	1.003083	0.2418857
PGARCH (1,1)	1.851597	**96.52826**	4.897602	1.00824	0.239865
PSE Composite Index					
GARCH (1,1)	1.810068	99.04375	5.080439	1.226183	0.2581086
EGARCH (1,1)	**1.785333**	**97.08529**	**4.988173**	**1.210813**	**0.2488187**
TGARCH (1,1)	1.81548	97.48082	5.052796	1.214231	0.2553075
GARCH-M (1,1)	1.808635	99.05043	5.074122	1.226355	0.2574671
PGARCH (1,1)	1.806505	97.27733	5.017069	1.213596	0.2517099

The table presents results from estimating regressions of volatility for each model and market. Columns indicate the particular loss functions, while the rows show corresponding volatility models under the selected markets. Numbers in bold demonstrate the minimum forecast error (preferred model)

A general conclusion for the daily forecasting results is that in most circumstances, the asymmetric models provide a smaller loss function than the symmetric models. Based on the error measures no specific model emerges as unconditionally best. Yet, in the presence of EGARCH, asymmetric models seem to outperform, especially in the developed markets, which contradicts the findings of Liu et al. (2009) to some extent. As asymmetric models reduce the forecast errors in emerging markets, the findings are relatively consistent and conclusive that asymmetric models perform best compared to the symmetric models. The conclusion is that asymmetric models provides smaller loss functions than symmetric models in some markets but symmetric models have no clear superiority for daily return series among the ten Asian markets, except for the recursive GARCH and GARCH-M models in the SSE Index. Therefore, according to the provided results, asymmetric models should be the best choice for market participants regardless of their degree of risk preference.

Table 5 presents the recursive forecasting results for the weekly return series, and one can see that the values in loss functions are higher compared to the daily forecasts, except for the MAPE, which is expected since it provides percentage errors. For the JCI index, the EGARCH model clearly outperforms the rest based on the four out of five loss functions. For the NIKKEI, STI, SSE and PSE indices, the EGARCH model is still favorable since it provides the smallest errors in MAE, RMSE and MSE error statistics, except for the QLIKE in all four cases. On the other hand, the HANG SENG Index is dominated by the TGARCH model, which provides the lowest values in all error statistics, consistent with the study done by Liu and Morley (2009). The remaining four indices are quite inconclusive, having no single volatility model that is preferred based on all five error statistics. However, focusing on the KLCI index, GARCH-M outperforms the rest under the MAE, RMSE and MSE error functions, with the GARCH model being best under the remaining two: an outcome which contradicts the study by Wong and Kok (2005), yet supports the findings of Brailsford and Faff (1996). The best forecasting model for Thailand’s SET index is PGARCH under the RMSE, QLIKE and MSE loss functions, EGARCH under MAE, and TGARCH under MAPE which is in line with the findings of Wong and Kok (2005). The SSE and TAIEX indices are inconclusive, with both the symmetric and the asymmetric models having superiority.

**Table 5:** Comparison of recursive forecast performance measures for weekly return series

	MAE	MAPE	RMSE	QLIKE	MSE
**Nikkei Index**					
GARCH (1,1)	10.24704195	109.7288047	34.60629227	3.197106	11.9759546
EGARCH (1,1)	**9.852790417**	110.9811024	**34.44267618**	3.165366	**11.8629794**
TGARCH (1,1)	10.2146914	**104.1148664**	34.87318235	**3.120967**	12.1613884
GARCH-M (1,1)	10.24704195	109.7288047	34.60629227	3.197106	11.9759546
PGARCH (1,1)	10.16925746	105.1745571	34.73206302	3.141197	12.0631620
Hang Seng Index					
GARCH (1,1)	9.32547705	97.54144908	20.61230543	3.067299	4.24867135
EGARCH (1,1)	9.339625916	96.69445478	20.37137611	3.063201	4.14992964
TGARCH (1,1)	**9.264218226**	**94.57426436**	**20.36580757**	**3.047739**	**4.14766118**
GARCH-M (1,1)	9.320921302	97.77387765	20.63026612	3.068798	4.2560788
PGARCH (1,1)	9.429220346	94.91954197	20.45221065	3.058514	4.18292920
Straits Times Index					
GARCH (1,1)	5.252157757	96.08916503	12.86998243	2.206019	1.65636447
EGARCH (1,1)	4.758979982	**94.10409623**	**12.51704479**	2.201988	**1.56676410**
TGARCH (1,1)	**4.689119989**	95.41533598	12.5355122	**2.201942**	1.57139066
GARCH-M (1,1)	5.245712123	96.39410246	12.86569795	2.205946	1.65526183
PGARCH (1,1)	4.739728191	94.80038753	12.54920083	2.204196	1.57482441
Set Index					
GARCH (1,1)	8.426017081	99.57559993	30.5917542	2.759791	9.35855424
EGARCH (1,1)	**8.256817365**	101.2233018	30.37921097	2.744206	9.22896459
TGARCH (1,1)	8.717362695	**98.15766856**	30.83602776	2.759911	9.50860607
GARCH-M (1,1)	8.423303418	99.80803376	30.59025658	2.761036	9.35763797
PGARCH (1,1)	8.113879608	100.6702069	**30.33050086**	**2.738054**	**9.19939282**
Kuala Lumpur Composite Index					
GARCH (1,1)	3.288348242	**102.4169325**	7.19721938	**1.843497**	0.51799966
EGARCH (1,1)	3.45261819	104.9358843	7.291681023	1.870931	0.53168612
TGARCH (1,1)	3.365769532	104.065921	7.229090199	1.879609	0.52259745
GARCH-M (1,1)	**3.285864066**	102.6715916	**7.196365017**	1.844444	**0.51787669**
PGARCH (1,1)	3.912468272	105.0466439	8.171850554	1.915069	0.66779141
Jakarta Composite Index					
GARCH (1,1)	10.62651772	104.5819448	28.38330434	2.925983	8.05611965
EGARCH (1,1)	**10.58209705**	105.0888898	**28.20886416**	**2.89873**	**7.95740017**
TGARCH (1,1)	10.99610195	103.1326459	28.63523156	2.924834	8.19976486
GARCH-M (1,1)	10.61432225	104.7936276	28.37364648	2.926143	8.05063814
PGARCH (1,1)	11.06978711	**102.7527687**	28.68031894	2.919125	8.22560694
SSE Composite Index					
GARCH (1,1)	14.97503352	94.91533057	26.8523909	**3.313693**	7.21050897
EGARCH (1,1)	**14.49030711**	97.06112885	**26.15783872**	3.316308	**6.84232526**
TGARCH (1,1)	15.15766964	96.24289017	27.10123656	3.321227	7.34477023
GARCH-M (1,1)	15.14179153	**94.79130681**	27.04246949	3.314682	7.31295156
PGARCH (1,1)	15.76411983	98.26341017	28.41487602	3.337693	8.07405179
Taiex Index					
GARCH (1,1)	**6.951689345**	96.35269128	12.59628114	**2.684637**	1.58666298
EGARCH (1,1)	7.060191741	96.55368882	12.64390506	2.693367	1.59868335
TGARCH (1,1)	6.986236118	**94.10762126**	**12.50567897**	2.691082	**1.56392006**
GARCH-M (1,1)	6.972881414	96.6849187	12.61696945	2.686138	1.59187918
PGARCH (1,1)	7.114754507	99.21654335	12.9244641	2.703149	1.67041772
Kospi Index					
GARCH (1,1)	8.946319583	97.52324323	26.6996366	2.688929	7.12870594
EGARCH (1,1)	**8.678639681**	102.4499881	27.0854921	2.706622	7.33623882
TGARCH (1,1)	9.022069322	**96.89577285**	26.48031987	2.689281	7.01207340
GARCH-M (1,1)	8.917973926	97.54169095	26.66787562	2.688099	7.11175590
PGARCH (1,1)	8.846015166	98.58714164	**26.43323642**	**2.686089**	**6.98715987**
PSE Composite Index					
GARCH (1,1)	8.767170108	94.1165022	21.82350589	2.854801	4.76265409
EGARCH (1,1)	**8.364475703**	97.36601358	**21.61199648**	2.851791	**4.67078392**
TGARCH (1,1)	8.78391413	**91.68777522**	21.6559497	2.852224	4.68980157
GARCH-M (1,1)	8.759685864	94.16410237	21.81974201	2.854756	4.76101141
PGARCH (1,1)	8.799686403	92.48756345	21.77040975	**2.848462**	4.73950740

The table presents results from estimating regressions of volatility for each model and market. Columns indicate the particular loss functions, while the rows show corresponding volatility models under the selected markets. Numbers in bold demonstrate the minimum forecast error (preferred model)

Table 6 shows the rolling window forecasts for weekly series, which is slightly different compared to recursive forecast results. Asymmetric models have clear superiority for the NIKKEI, HANG SENG, SET, JCI, TAIEX and PSE indices. These results are consistent with those of Awartani and Corradi (2005) and Evans and McMillan (2007), which reveal supportive evidence for asymmetric GARCH models producing more accurate predictions in volatility forecasting. The results also display that the asymmetry effect should be considered by investors when they deal with the Asian markets mentioned above. Furthermore, the STI, SSE and KOSPI indices present mixed results where volatility prediction can be examined by employing either symmetric or asymmetric GARCH models. The KLCI index is dominated by predictions of symmetric models which does not support the findings of Balaban et al. (2006) where they recommended asymmetric models for the Malaysian stock market. According to these results, the Malaysian stock market does not seem to follow an asymmetric volatility pattern, and therefore investors can rely on the predictions of symmetric GARCH models in the medium term.

**Table 6:** Comparison of rolling window forecast performance measures for weekly return series

	MAE	MAPE	RMSE	QLIKE	MSE
Nikkei Index					
GARCH (1,1)	10.23903752	115.3234282	34.6895999	3.228608	12.0336834
EGARCH (1,1)	**10.02960406**	110.4678963	**34.4978507**	3.15849	**11.9010170**
TGARCH (1,1)	10.18490051	**105.5480224**	34.87293549	**3.118403**	12.1612163
GARCH-M (1,1)	10.50922895	111.6392732	34.6563506	3.665166	NA
PGARCH (1,1)	10.16097164	107.9813858	35.27289295	3.133957	12.4417697
Hang Seng Index					
GARCH (1,1)	9.347053394	97.75967218	20.65139571	3.068174	4.26480144
EGARCH (1,1)	**9.118997728**	96.74101014	**20.15971826**	3.052794	**4.06414240**
TGARCH (1,1)	9.219939215	**95.76319774**	20.47479875	**3.047555**	4.19217383
GARCH-M (1,1)	9.338574572	97.99313533	20.66608513	3.069463	4.27087074
PGARCH (1,1)	9.164710922	95.99475087	20.36375296	3.04891	4.14682434
Straits Times Index					
GARCH (1,1)	5.112268729	**97.89894806**	12.80623319	2.196915	1.63999608
EGARCH (1,1)	4.721304268	100.264789	12.55809201	2.212307	1.57705675
TGARCH (1,1)	**4.615153369**	100.1671187	**12.53723108**	2.205709	**1.57182163**
GARCH-M (1,1)	5.106817953	98.18061964	12.80253485	**2.196883**	1.63904898
PGARCH (1,1)	4.701966549	99.08547731	12.56324032	2.203582	1.57835007
Set Index					
GARCH (1,1)	8.559501846	100.7626483	30.71614474	2.795522	9.43481547
EGARCH (1,1)	**8.319327042**	100.9050598	**30.52793742**	**2.773872**	**9.31954963**
TGARCH (1,1)	8.994764986	**99.48470097**	31.0105933	2.818087	9.61656896
GARCH-M (1,1)	8.548545804	100.7025553	30.69755824	2.792438	9.42340081
PGARCH (1,1)	8.386810531	105.4101086	30.63860185	2.831149	9.38723923
Kuala Lumpur Composite Index					
GARCH (1,1)	3.297103816	**102.2603225**	7.211087021	**1.841896**	0.51999776
EGARCH (1,1)	3.369799	105.3390351	7.228977783	1.866069	0.52258119
TGARCH (1,1)	3.383946928	107.5000312	7.312593617	1.888627	0.53474025
GARCH-M (1,1)	**3.293692809**	102.4692105	**7.208588101**	1.842553	**0.51963742**
PGARCH (1,1)	3.42816284	107.3560786	7.37594915	1.890691	0.54404625
Jakarta Composite Index					
GARCH (1,1)	10.73660772	103.6236215	28.36464407	2.930556	8.04553033
EGARCH (1,1)	**10.69562249**	103.0196581	**28.11420106**	**2.908711**	**7.90408301**
TGARCH (1,1)	11.12750968	**101.3239555**	28.4687193	2.931287	8.10467978
GARCH-M (1,1)	10.75247188	103.8831999	28.3614357	2.932319	8.04371035
PGARCH (1,1)	11.32325464	101.9060386	28.57454283	2.936134	8.16504497
SSE Composite Index					
GARCH (1,1)	13.9018026	104.3783375	26.30870349	3.324486	6.92147879
EGARCH (1,1)	**13.40776975**	105.1831596	**25.90241788**	3.318551	**6.70935252**
TGARCH (1,1)	13.72463722	105.7850418	26.2520718	3.324384	6.89171273
GARCH-M (1,1)	13.89610633	**103.9302372**	26.22439829	**3.318003**	6.87719065
PGARCH (1,1)	13.85895519	105.9204464	26.49834026	3.33063	7.02162036
Taiex Index					
GARCH (1,1)	6.929810747	97.44531374	12.64146631	2.680249	1.59806670
EGARCH (1,1)	6.94199152	96.96124223	12.63376083	**2.675296**	1.59611912
TGARCH (1,1)	**6.908039017**	**96.28216513**	**12.56213378**	2.685641	**1.57807205**
GARCH-M (1,1)	6.941396055	97.80271546	12.65337317	2.682452	1.60107852
PGARCH (1,1)	7.222127158	98.81056654	13.069708	2.694263	1.70817267
Kospi Index					
GARCH (1,1)	9.076358544	95.78283164	26.6779072	2.681991	7.11710732
EGARCH (1,1)	**8.977506471**	95.28897378	**26.08869043**	2.693891	**6.80619768**
TGARCH (1,1)	9.273876861	**93.62840096**	26.4485096	2.687859	6.99523660
GARCH-M (1,1)	9.062519328	95.88973008	26.69104258	**2.680772**	7.12411753
PGARCH (1,1)	9.229063314	94.09587094	26.32159032	2.698456	6.92826117
PSE Composite Index					
GARCH (1,1)	8.781616991	93.4202291	21.82425782	2.841792	4.76298229
EGARCH (1,1)	**8.466831256**	95.19936853	**21.54344241**	**2.831211**	**4.64119910**
TGARCH (1,1)	8.778769695	92.38548657	21.69140553	2.845177	4.70517073
GARCH-M (1,1)	8.749702644	93.51903077	21.79605048	2.838839	4.75067816
PGARCH (1,1)	9.381787835	**91.39264257**	22.20446446	2.852795	4.93038242

The table presents results from estimating regressions of volatility for each model and market. Columns indicate the particular loss functions, while the rows show corresponding volatility models under the selected markets. Numbers in bold demonstrate the minimum forecast error (preferred model)

Table 7 reports the monthly out-of-sample forecasting results based on the recursive method. Statistical values increase with the reducing frequency compared to daily and weekly time frames, which is expected, except for the percentage-based loss function MAPE. The results are very surprising compared to daily and weekly outcomes. The only superiority for asymmetric models is reported from the STI index, where the PGARCH and EGARCH models are recommended based on the MAPE and remaining loss functions respectively. The NIKKEI, HANG SENG, SSE, KOSPI and PSE models indicate mixed results and are fairly incomplete in terms of the most preferred model, yet either symmetric or asymmetric models can be conducted for prediction depending on the selected loss functions. Still, it can be said based on the estimated results that these five markets are indecisive and neither symmetric nor asymmetric models dominate each other which supports the earlier work of Ng and McAleer (2004). On the other hand, symmetric models dominate in the SET and TAIEX indices except for the MAPE statistic, which suggests EGARCH superiority. The smallest error values are provided by the symmetric GARCH models under all statistics for the KLCI and JCI indices which is in line with the findings of Minkah (2007) and Lee et al. (2017). Thus, the GARCH and GARCH-M models can be the best forecast models in this two markets for either econometricians or other market participants.

**Table 7:** Comparison of recursive forecast performance measures for monthly return series

	MAE	MAPE	RMSE	QLIKE	MSE
Nikkei Index					
GARCH (1,1)	36.13937171	108.9165511	71.39343577	4.474199	50.9702267
EGARCH (1,1)	**33.74478972**	126.4161671	71.72815663	4.51878	51.4492845
TGARCH (1,1)	36.42051006	110.5662039	72.60488221	4.474801	52.7146892
GARCH-M (1,1)	36.11342058	108.2973356	**71.18787899**	**4.467483**	**50.6771411**
PGARCH (1,1)	43.50617923	**90.5801923**	79.97545547	4.53929	63.9607347
Hang Seng Index					
GARCH (1,1)	44.11303339	106.3745122	75.4767641	**4.515765**	56.9674192
EGARCH (1,1)	44.50939769	108.7900812	**74.96431533**	4.552106	**56.1964857**
TGARCH (1,1)	44.87925045	105.8944785	76.3272369	4.528689	58.2584709
GARCH-M (1,1)	**43.965541**	109.0935159	75.17715281	4.536132	56.5160430
PGARCH (1,1)	63.46523175	**94.40515162**	83.0831442	4.763705	69.0280885
Straits Times Index					
GARCH (1,1)	26.39027302	92.42545973	46.0282317	3.805429	21.1859811
EGARCH (1,1)	**24.17079656**	86.53822773	**41.85116394**	**3.774182**	**17.5151992**
TGARCH (1,1)	24.84527773	85.81235534	43.62661678	3.767626	19.0328169
GARCH-M (1,1)	26.38496415	92.90026529	45.97301665	3.812358	21.1351826
PGARCH (1,1)	27.50161943	**83.91430967**	49.34098297	3.788462	24.3453260
Set Index					
GARCH (1,1)	**40.14294886**	98.66219174	**114.4438535**	4.289066	**130.973956**
EGARCH (1,1)	46.57016969	**87.34734209**	115.5980647	4.351424	133.629125
TGARCH (1,1)	41.31889381	96.84485635	116.1339308	4.291495	134.870898
GARCH-M (1,1)	40.47797495	97.68903105	114.4799052	**4.28838**	131.056486
PGARCH (1,1)	40.16653678	96.68829158	115.8919697	4.309496	134.309486
Kuala Lumpur Composite Index					
GARCH (1,1)	15.81671405	**100.8019022**	29.94179975	**3.378925**	8.96511372
EGARCH (1,1)	15.76343036	104.3824237	29.29805807	3.387469	**8.58376206**
TGARCH (1,1)	16.39680944	120.7636268	30.44154928	3.601556	9.26687922
GARCH-M (1,1)	**15.74525061**	102.3368336	**29.91456572**	3.386437	8.94881242
PGARCH (1,1)	16.5589291	117.4855398	31.24939783	3.556378	9.76524864
Jakarta Composite Index					
GARCH (1,1)	48.42643097	**89.62353775**	**123.4907233**	4.50241	**152.499587**
EGARCH (1,1)	66.8645489	95.93257192	139.8322435	4.594498	195.530563
TGARCH (1,1)	57.13590737	102.9657686	130.2570443	4.675225	169.668975
GARCH-M (1,1)	**48.30416115**	89.83505255	123.5078662	**4.497987**	152.54193
PGARCH (1,1)	58.74737026	96.2757173	143.843966	4.579117	206.910865
SSE Composite Index					
GARCH (1,1)	95.8644111	**114.1801706**	153.1306091	**5.198126**	234.489834
EGARCH (1,1)	94.32650292	128.5285823	**149.8592953**	5.285649	**224.578084**
TGARCH (1,1)	98.91509954	117.0330358	159.0790699	5.225212	253.061504
GARCH-M (1,1)	**93.09644502**	273.0684384	153.2074552	5.216335	234.725243
PGARCH (1,1)	100.3712522	120.0527793	164.7702377	5.232148	271.492312
Taiex Index					
GARCH (1,1)	**28.18671674**	95.04204526	**50.06840478**	**4.023104**	**25.0684515**
EGARCH (1,1)	43.19547421	**81.27518973**	52.2496524	4.259888	27.3002617
TGARCH (1,1)	31.25622181	86.49914406	50.84625876	4.044196	25.8534203
GARCH-M (1,1)	31.98645284	83.08716116	54.44363312	4.824353	NA
PGARCH (1,1)	34.09138681	90.51921831	55.12185896	4.120919	30.3841933
Kospi Index					
GARCH (1,1)	32.62617527	94.65923674	66.50816143	4.047954	44.2333553
EGARCH (1,1)	32.17000663	101.205364	66.51652596	4.058136	44.2444822
TGARCH (1,1)	34.7002436	99.77480809	69.42858055	4.091565	48.2032779
GARCH-M (1,1)	32.42825266	**92.66106591**	66.39169416	**4.027558**	44.0785705
PGARCH (1,1)	**29.39189557**	99.71261927	**59.43565374**	4.037928	**35.3259693**
PSE Composite Index					
GARCH (1,1)	33.78831738	90.44866151	70.43109114	4.30187	49.6053859
EGARCH (1,1)	36.61106192	98.29816079	71.03252277	4.392878	50.4561929
TGARCH (1,1)	38.06951705	**84.10260598**	71.83112695	4.336803	51.5971079
GARCH-M (1,1)	**33.5974511**	91.90064622	70.59149337	**4.301618**	49.8315893
PGARCH (1,1)	34.03357458	90.58543272	**70.10124654**	4.307639	**49.1418476**

The table presents results from estimating regressions of volatility for each model and market. Columns indicate the particular loss functions, while the rows show corresponding volatility models under the selected markets. Numbers in bold demonstrate the minimum forecast error (preferred model)

Based on the rolling window forecast results as presented in Table 8, asymmetric models are clearly superior in the NIKKEI and SET indices, while EGARCH models are the single superior type based on all statistics in the JCI index. This is very surprising, since the recursive method recommends symmetric GARCH models for the JCI index, whereas the rolling window method does not offer a recommendation at all. In addition, the GARCH and GARCH-M models dominate in the HANG SENG index, which supports the findings of Gokcan (2000), yet contradicts the studies of Liu and Morley (2009), and Sabiruzzaman et al. (2010) where they recommend the EGARCH and TGARCH models respectively for Hong Kong stock market returns. The remaining indices are indecisive and inconclusive in terms of the dominance of symmetric and asymmetric GARCH models, yet they support the work of Etac and Ceballos (2018).

**Table 8:** Comparison of rolling window forecast performance measures for monthly return series

	MAE	MAPE	RMSE	QLIKE	MSE
Nikkei Index					
GARCH (1,1)	35.68034204	137.6621932	71.37525994	4.607964	50.9442773
EGARCH (1,1)	**35.6192196**	110.5984806	**71.09597635**	**4.449931**	**50.5463785**
TGARCH (1,1)	36.62242496	114.3439591	73.04373464	4.471616	53.3538717
GARCH-M (1,1)	1.61E + 27	114.5584416	1.95E + 28	5.351015	3.82E + 56
PGARCH (1,1)	46.30306072	**105.7651175**	81.02921426	4.670946	65.6573356
Hang Seng Index					
GARCH (1,1)	44.26956718	**109.7175226**	76.87958946	**4.525215**	59.1047127
EGARCH (1,1)	44.10793906	115.8362886	76.01769424	4.610958	57.7868983
TGARCH (1,1)	45.59502803	111.4239774	77.32264709	4.568397	59.7879175
GARCH-M (1,1)	**43.80363089**	112.7806508	**75.93705209**	4.548415	**57.6643588**
PGARCH (1,1)	52.77150671	117.5579398	78.68502738	4.746715	61.9133353
Straits Times Index					
GARCH (1,1)	24.51726305	**93.17751469**	42.46318504	3.778342	18.0312208
EGARCH (1,1)	22.5504177	104.3194475	40.63369021	3.796456	16.5109678
TGARCH (1,1)	**22.04210235**	102.7597891	**38.33341071**	3.775107	**14.6945037**
GARCH-M (1,1)	24.49186442	94.26876325	43.00772655	3.784755	18.4966454
PGARCH (1,1)	24.00816988	99.11157724	43.16818931	**3.736314**	18.6349256
Set Index					
GARCH (1,1)	40.89568127	96.97461538	116.1304944	4.284368	134.862917
EGARCH (1,1)	42.99820562	**87.30635411**	113.8064589	4.358476	129.519100
TGARCH (1,1)	41.27529169	87.89284013	116.8322756	**4.275816**	136.497806
GARCH-M (1,1)	41.05914869	95.55166231	115.5835173	4.280834	133.595494
PGARCH (1,1)	**37.90849038**	95.48605127	**111.9304698**	4.326555	**125.284300**
Kuala Lumpur Composite Index					
GARCH (1,1)	**15.77922042**	**103.2035548**	30.07818857	**3.395053**	9.04697427
EGARCH (1,1)	15.94107972	104.8350858	**29.9386287**	3.413558	**8.96321488**
TGARCH (1,1)	16.99634379	114.2177454	31.27121235	3.529704	9.77888721
GARCH-M (1,1)	1.02E + 242	104.8220037	NA	10.34143	NA
PGARCH (1,1)	22.52956236	124.1088923	34.18635538	3.846813	11.6870689
Jakarta Composite Index					
GARCH (1,1)	46.0127606	89.11073762	122.7407655	4.421782	150.652955
EGARCH (1,1)	**42.60991903**	**88.74935183**	**116.8132784**	**4.295547**	**136.453420**
TGARCH (1,1)	50.18068336	92.53907147	127.6355682	4.433328	162.908382
GARCH-M (1,1)	47.04107957	90.91205072	123.2588292	4.449774	151.927389
PGARCH (1,1)	54.7163157	95.28763404	142.990259	4.486811	204.462141
SSE Composite Index					
GARCH (1,1)	83.77736342	**124.8769572**	139.3492393	**5.21587**	194.182105
EGARCH (1,1)	83.11516659	147.1610836	136.9202249	5.482313	187.471479
TGARCH (1,1)	80.09052779	206.1656405	139.5677941	5.947184	194.791691
GARCH-M (1,1)	7.50E + 38	130.2258094	9.09E + 39	5.879085	8.26E + 79
PGARCH (1,1)	**79.60418389**	130.9162032	**134.4769281**	5.219584	**180.840442**
Taiex Index					
GARCH (1,1)	**28.29841929**	96.42357262	50.47603031	4.018177	25.4782963
EGARCH (1,1)	33.05684801	**83.84097093**	53.0489444	4.029078	28.1419050
TGARCH (1,1)	29.12683151	92.9883831	**50.01473118**	4.028996	**25.0147333**
GARCH-M (1,1)	1.39E + 197	93.71602343	NA	12.60411	NA
PGARCH (1,1)	28.30199991	89.98944316	50.28092746	**3.9715**	25.2817166
Kospi Index					
GARCH (1,1)	**30.91354564**	109.2698937	**65.92265188**	**4.006989**	**43.4579603**
EGARCH (1,1)	34.37528301	99.65449925	69.43052129	4.070387	48.2059728
TGARCH (1,1)	33.64263426	109.9297935	70.90057768	4.017745	50.2689191
GARCH-M (1,1)	707.0296796	106.3544693	2251.795946	4.454703	50.7058498
PGARCH (1,1)	32.64005915	**99.78512706**	67.46846057	4.027628	45.5199317
PSE Composite Index					
GARCH (1,1)	**33.82456354**	97.96613715	**71.18282651**	**4.37822**	**50.6699479**
EGARCH (1,1)	39.1332937	92.95452889	73.20748989	4.391089	53.5933657
TGARCH (1,1)	42.35321892	86.68376403	80.85382678	4.429187	65.3734130
GARCH-M (1,1)	2.23E + 304	248.0119527	NA	113.9237	NA
PGARCH (1,1)	39.36423634	**86.54202952**	72.57175236	4.427396	52.6665924

The table presents results from estimating regressions of volatility for each model and market. Columns indicate the particular loss functions, while the rows show corresponding volatility models under the selected markets. Numbers in bold demonstrate the minimum forecast error (preferred model)

Tables from 9, 10, 11, 12, 13, 14, 15, 16, 17 and 18 report pairwise Diebold and Mariano test results for a further evaluation of the performance in selected forecasting models for each selected index. In the tables, DM(A) and DM(S) indicate DM test statistics based on the absolute-error loss and the squared-error loss, respectively. Their corresponding *p*-values are also attached for each statistic to show the level of significance.

**Table 9:** DM test statistics and *p* values for NIKKEI Index

Forecast 1 vs Forecast 2	DM(A)	< > *p* values	> *p* values	< *p* values	DM(S)	< > *p* values	> *p* values	< *p* values	DM(A)	< > *p* values	> *p* values	< *p* values	DM(S)	< > *p* values	> *p* values	< *p* values
Recursive daily									Rolling window daily							
GARCH vs EGARCH	4.9749	0.0000	1.0000	0.0000	0.4536	0.6502	0.6749	0.3251	5.4186	0.0000	1.0000	0.0000	0.8386	0.4018	0.7991	0.2009
GARCH vs TGARCH	0.5074	0.6119	0.6940	0.3060	1.3588	0.1743	0.9128	0.0872	1.0137	0.3108	0.8446	0.1554	1.5337	0.1252	0.9374	0.0626
GARCH vs MGARCH	5.1672	0.0000	1.0000	0.0000	− 0.2520	0.8011	0.4005	0.5995	3.0167	0.0026	0.9987	0.0013	− 0.6530	0.5138	0.2569	0.7431
GARCH vs PGARCH	3.6168	0.0003	0.9998	0.0002	0.7550	0.4503	0.7748	0.2252	2.7372	0.0062	0.9969	0.0031	0.8376	0.4023	0.7988	0.2012
EGARCH vs TGARCH	− 4.2163	0.0000	0.0000	0.0000	0.6318	0.5276	0.7362	0.2638	− 4.7943	0.0000	0.0000	1.0000	0.3104	0.7562	0.6219	0.3781
EGARCH vs MGARCH	− 4.9436	0.0000	0.0000	0.0000	− 0.4551	0.6490	0.3245	0.6755	− 5.3951	0.0000	0.0000	1.0000	− 0.8458	0.3977	0.1989	0.8011
EGARCH vs PGARCH	− 3.0604	0.0022	0.0011	0.9989	0.2336	0.8153	0.5923	0.4077	− 4.2289	0.0000	0.0000	1.0000	− 0.5114	0.6091	0.3045	0.6955
TGARCH vs MGARCH	− 0.4372	0.6620	0.3310	0.6690	− 1.3515	0.1766	0.0883	0.9117	− 0.9531	0.3406	0.1703	0.8297	− 1.5231	0.1278	0.0639	0.9361
TGARCH vs PGARCH	2.9876	0.0028	0.9986	0.0014	− 0.7352	0.4623	0.2311	0.7689	2.0369	0.0418	0.9791	0.0209	− 0.7262	0.4678	0.2339	0.7661
MGARCH vs PGARCH	3.5715	0.0004	0.9998	0.0002	0.7557	0.4499	0.7750	0.2250	2.7020	0.0069	0.9965	0.0035	0.8452	0.3981	0.8010	0.1990
Recursive weekly									Rolling window weekly							
GARCH vs EGARCH	1.4580	0.1454	0.9273	0.0727	0.2791	0.7803	0.6099	0.3901	0.7815	0.4348	0.7826	0.2174	0.2971	0.7665	0.6168	0.3832
GARCH vs TGARCH	0.1565	0.8757	0.5621	0.4379	− 0.4182	0.6759	0.3380	0.6620	0.2404	0.8101	0.5950	0.4050	− 0.3150	0.7529	0.3764	0.6236
GARCH vs MGARCH	0.2526	0.8007	0.5997	0.4003	0.0604	0.9518	0.5241	0.4759	NA	NA	NA	NA	NA	NA	NA	NA
GARCH vs PGARCH	0.4682	0.6398	0.6801	0.3199	− 0.3393	0.7345	0.3673	0.6327	0.3219	0.7476	0.6262	0.3738	− 0.7486	0.4544	0.2272	0.7728
EGARCH vs TGARCH	− 0.8759	0.3814	0.1907	0.8093	− 0.4154	0.6780	0.3390	0.6610	− 0.3859	0.6997	0.3499	0.6501	− 0.3588	0.7199	0.3599	0.6401
EGARCH vs MGARCH	− 1.4580	0.1454	0.0727	0.9273	– 0.2791	0.7803	0.3901	0.6099	NA	NA	NA	NA	NA	NA	NA	NA
EGARCH vs PGARCH	− 0.8960	0.3706	0.1853	0.8147	– 0.3509	0.7258	0.3629	0.6371	− 0.2987	0.7653	0.3826	0.6174	− 0.6108	0.5416	0.2708	0.7292
TGARCH vs MGARCH	− 0.1565	0.8757	0.4379	0.5621	0.4182	0.6759	0.6620	0.3380	NA	NA	NA	NA	NA	NA	NA	NA
TGARCH vs PGARCH	0.3580	0.7205	0.6398	0.3602	0.4799	0.6315	0.6843	0.3157	0.3052	0.7603	0.6199	0.3801	− 1.3085	0.1912	0.0956	0.9044
MGARCH vs PGARCH	0.4682	0.6398	0.6801	0.3199	− 0.3393	0.7345	0.3673	0.6327	NA	NA	NA	NA	NA	NA	NA	NA
Recursive monthly									Rolling window monthly							
GARCH vs EGARCH	2.6604	0.0088	0.9956	0.0044	1.5994	0.1121	0.9439	0.0561	0.2203	0.8259	0.5870	0.4130	1.1821	0.2392	0.8804	0.1196
GARCH vs TGARCH	− 0.3078	0.7587	0.3793	0.6207	− 1.0520	0.2946	0.1473	0.8527	− 0.7750	0.4396	0.2198	0.7802	− 0.9053	0.3668	0.1834	0.8166
GARCH vs MGARCH	0.6667	0.5060	0.7470	0.2530	1.1985	0.2327	0.8837	0.1163	− 1.0005	0.3187	0.1594	0.8406	− 1.0000	0.3190	0.1595	0.8405
GARCH vs PGARCH	− 3.3190	0.0011	0.0006	0.9994	− 1.2023	0.2312	0.1156	0.8844	− 4.3606	0.0000	0.0000	1.0000	− 1.5573	0.1216	0.0608	0.9392
EGARCH vs TGARCH	− 2.1601	0.0326	0.0163	0.9837	− 1.4754	0.1425	0.0712	0.9288	− 0.8382	0.4033	0.2017	0.7983	− 1.3415	0.1819	0.0910	0.9090
EGARCH vs MGARCH	− 2.6577	0.0088	0.0044	0.9956	− 1.5289	0.1287	0.0643	0.9357	− 1.0005	0.3188	0.1594	0.8406	− 1.0000	0.3190	0.1595	0.8405
EGARCH vs PGARCH	− 3.5115	0.0006	0.0003	0.9997	− 1.2710	0.2059	0.1030	0.8970	− 4.2538	0.0000	0.0000	1.0000	− 1.6875	0.0937	0.0469	0.9531
TGARCH vs MGARCH	0.3300	0.7419	0.6291	0.3709	1.2090	0.2286	0.8857	0.1143	− 1.0005	0.3187	0.1594	0.8406	− 1.0000	0.3190	0.1595	0.8405
TGARCH vs PGARCH	− 3.2408	0.0015	0.0007	0.9993	− 1.1771	0.2411	0.1205	0.8795	− 4.3562	0.0000	0.0000	1.0000	− 1.7234	0.0870	0.0435	0.9565
MGARCH vs PGARCH	− 3.3163	0.0012	0.0006	0.9994	− 1.2241	0.2229	0.1114	0.8886	1.0005	0.3187	0.8406	0.1594	1.0000	0.3190	0.8405	0.1595

1.The columns labelled DM(A) and DM(S) contain t-statistic based on absolute and squared prediction errors, respectively. 2. The null hypothesis of DM-test is that of equal predictive ability of the two models; a significantly positive (negative) t-statistics indicates the benchmark model is dominated by (dominates) the corresponding model

**Table 10:** DM test statistics and *p* values for STI Index

Forecast 1 vs Forecast 2	DM(A)	< > *p* values	> *p* values	< *p* values	DM(S)	< > *p* values	> *p* values	< *p* values	DM(A)	< > *p* values	> *p* values	< *p* values	DM(S)	< > *p* values	> *p* values	< *p* values
Recursive daily									Rolling window daily							
GARCH vs EGARCH	0.3525	0.7245	0.6377	0.3623	0.1201	0.9044	0.5478	0.4522	0.4491	0.6534	0.6733	0.3267	− 0.0945	0.9247	0.4624	0.5376
GARCH vs TGARCH	− 1.4827	0.1383	0.0691	0.3060	− 0.2078	0.8354	0.4177	0.5823	− 0.0585	0.9534	0.4767	0.5233	− 0.0585	0.9534	0.4767	0.5233
GARCH vs MGARCH	− 1.0608	0.2889	0.1444	0.8556	− 0.9189	0.3582	0.1791	0.8209	0.6141	0.5392	0.7304	0.2696	− 0.4847	0.6279	0.3140	0.6860
GARCH vs PGARCH	− 3.7518	0.0002	0.0001	0.9999	− 1.5502	0.1212	0.0606	0.9394	− 2.6969	0.0070	0.0035	0.9965	− 1.0686	0.2854	0.1427	0.8573
EGARCH vs TGARCH	− 2.3466	0.0190	0.0095	0.9905	− 0.7116	0.4768	0.2384	0.7616	− 0.8121	0.4168	0.2084	0.7916	0.4150	0.6782	0.6609	0.3391
EGARCH vs MGARCH	− 0.3780	0.7054	0.3527	0.6473	− 0.1460	0.8839	0.4420	0.5580	− 0.3957	0.6924	0.3462	0.6538	0.0732	0.9416	0.5292	0.4708
EGARCH vs PGARCH	− 4.3895	0.0000	0.0000	1.0000	− 1.8886	0.0591	0.0295	0.9705	− 3.7728	0.0002	0.0001	0.9999	− 2.0674	0.0388	0.0194	0.9806
TGARCH vs MGARCH	1.4168	0.1567	0.9217	0.0783	0.1603	0.8727	0.5637	0.4363	0.1099	0.9125	0.5438	0.4562	− 0.1165	0.9073	0.4536	0.5464
TGARCH vs PGARCH	− 2.9375	0.0033	0.0017	0.9983	− 1.8105	0.0704	0.0352	0.9648	− 3.2226	0.0013	0.0006	0.9994	− 2.5626	0.0104	0.0052	0.9948
MGARCH vs PGARCH	− 3.6890	0.0002	0.0001	0.9999	− 1.4817	0.1385	0.0693	0.9307	− 2.7073	0.0068	0.0034	0.9966	− 1.0102	0.3125	0.1563	0.8437
Recursive weekly									Rolling window weekly							
GARCH vs EGARCH	3.5782	0.0004	0.9998	0.0002	0.9864	0.3244	0.8378	0.1622	3.1445	0.0018	0.9991	0.0009	0.8500	0.3957	0.8021	0.1979
GARCH vs TGARCH	4.5686	0.0000	1.0000	0.0000	0.9733	0.3309	0.8346	0.1654	4.2335	0.0000	1.0000	0.0000	0.8965	0.3704	0.8148	0.1852
GARCH vs MGARCH	2.2573	0.0244	0.9878	0.0122	1.3761	0.1694	0.9153	0.0847	1.3569	0.1754	0.9123	0.0877	0.7902	0.4298	0.7851	0.2149
GARCH vs PGARCH	3.9430	0.0001	1.0000	0.0000	0.9035	0.3667	0.8166	0.1834	3.3647	0.0008	0.9996	0.0004	0.8062	0.4205	0.7897	0.2103
EGARCH vs TGARCH	1.5739	0.1162	0.9419	0.0581	− 0.3291	0.7423	0.3711	0.6289	3.1282	0.0019	0.9991	0.0009	0.5204	0.6031	0.6985	0.3015
EGARCH vs MGARCH	− 3.5338	0.0004	0.0002	0.9998	− 0.9694	0.3328	0.1664	0.8336	− 3.1143	0.0020	0.0010	0.9990	− 0.8404	0.4011	0.2005	0.7995
EGARCH vs PGARCH	0.4972	0.6193	0.6903	0.3097	− 0.7285	0.4667	0.2333	0.7667	0.7660	0.4440	0.7780	0.2220	− 0.1513	0.8798	0.4399	0.5601
TGARCH vs MGARCH	− 4.5163	0.0000	0.0000	1.0000	− 0.9552	0.3399	0.1700	0.8300	− 4.2010	0.0000	0.0000	1.0000	− 0.8871	0.3755	0.1877	0.8123
TGARCH vs PGARCH	− 1.9641	0.0501	0.0250	0.9750	− 0.5849	0.5589	0.2794	0.7206	− 3.6698	0.0003	0.0001	0.9999	− 1.4721	0.1417	0.0708	0.9292
MGARCH vs PGARCH	3.8935	0.0001	0.9999	0.0001	0.8864	0.3758	0.8121	0.1879	3.3341	0.0009	0.9995	0.0005	0.7966	0.4261	0.7870	0.2130
Recursive monthly									Rolling window monthly							
GARCH vs EGARCH	1.1500	0.2526	0.8737	0.1263	0.8651	0.3889	0.8056	0.1944	0.9552	0.3415	0.8292	0.1708	0.3530	0.7247	0.6376	0.3624
GARCH vs TGARCH	1.6585	0.1000	0.9500	0.0500	1.2515	0.2134	0.8933	0.1067	1.6524	0.1013	0.9494	0.0506	1.3344	0.1848	0.9076	0.0924
GARCH vs MGARCH	0.0109	0.9913	0.5043	0.4957	0.0475	0.9622	0.5189	0.4811	0.0645	0.9487	0.5257	0.4743	− 0.7654	0.4457	0.2228	0.7772
GARCH vs PGARCH	− 0.7764	0.4392	0.2196	0.7804	− 1.0363	0.3023	0.1512	0.8488	0.2429	0.8086	0.5957	0.4043	− 0.1384	0.8902	0.4451	0.5549
EGARCH vs TGARCH	− 0.4819	0.6308	0.3154	0.6846	− 0.4300	0.6681	0.3340	0.6660	0.5031	0.6159	0.6921	0.3079	0.6753	0.5009	0.7495	0.2505
EGARCH vs MGARCH	− 1.0997	0.2738	0.1369	0.8631	− 0.8436	0.4007	0.2004	0.7996	− 0.9150	0.3622	0.1811	0.8189	− 0.4591	0.6470	0.3235	0.6765
EGARCH vs PGARCH	− 1.7992	0.0747	0.0374	0.9626	− 1.8376	0.0688	0.0344	0.9656	− 1.2471	0.2150	0.1075	0.8925	− 1.6301	0.1059	0.0530	0.9470
TGARCH vs MGARCH	− 1.1880	0.2374	0.1187	0.8813	− 0.8063	0.4218	0.2109	0.7891	− 1.4341	0.1544	0.0772	0.9228	− 1.3391	0.1833	0.0916	0.9084
TGARCH vs PGARCH	− 2.2605	0.0257	0.0129	0.9871	− 1.8509	0.0668	0.0334	0.9666	− 1.8609	0.0654	0.0327	0.9673	− 1.4788	0.1420	0.0710	0.9290
MGARCH vs PGARCH	− 0.6760	0.5005	0.2502	0.7498	− 0.9522	0.3431	0.1715	0.8285	0.2101	0.8339	0.5830	0.4170	− 0.0308	0.9755	0.4878	0.5122

1. The columns labelled DM(A) and DM(S) contain t-statistic based on absolute and squared prediction errors, respectively. 2. The null hypothesis of DM-test is that of equal predictive ability of the two models; a significantly positive (negative) t-statistics indicates the benchmark model is dominated by (dominates) the corresponding model

**Table 11:** DM test statistics and *p* values for Hang Seng Index

Forecast 1 vs Forecast 2	DM(A)	< > *p* value	> *p* value	< *p* value	DM(S)	< > *p* value	> *p* value	< *p* value	DM(A)	< > *p* value	> *p* value	< *p* value	DM(S)	< > *p* value	> *p* value	*p* value
Recursive daily									Rolling window daily							
GARCH vs EGARCH	3.0324	0.0024	0.9988	0.0012	2.9417	0.0033	0.9984	0.0016	4.3739	0.0000	1.0000	0.0000	2.6013	0.0093	0.9953	0.0047
GARCH vs TGARCH	2.7157	0.0067	0.9967	0.0033	2.7030	0.0069	0.9965	0.0035	4.2016	0.0000	1.0000	0.0000	2.5941	0.0095	0.9952	0.0048
GARCH vs MGARCH	2.8790	0.0040	0.9980	0.0020	2.0756	0.0380	0.9810	0.0190	2.5799	0.0099	0.9950	0.0050	1.9687	0.0491	0.9755	0.0245
GARCH vs PGARCH	1.3262	0.1849	0.0924	0.9076	2.3201	0.0204	0.9898	0.0102	4.9956	0.0000	1.0000	0.0000	2.7382	0.0062	0.9969	0.0031
EGARCH vs TGARCH	− 2.1728	0.0299	0.0149	0.9851	− 0.3778	0.7056	0.3528	0.6472	− 2.6507	0.0081	0.0040	0.9960	0.4495	0.6531	0.6735	0.3265
EGARCH vs MGARCH	− 2.9816	0.0029	0.0014	0.9986	2.8960	0.0038	0.0019	0.9981	− 4.3056	0.0000	0.0000	1.0000	− 2.4366	0.0149	0.0074	0.9926
EGARCH vs PGARCH	− 4.6601	0.0000	0.0000	1.0000	− 0.4472	0.6548	0.3274	0.6726	− 1.5784	0.1146	0.0573	0.9427	0.9052	0.3654	0.8173	0.1827
TGARCH vs MGARCH	− 2.6658	0.0077	0.0039	0.9961	− 2.7333	0.0063	0.0032	0.9968	− 4.2264	0.0000	0.0000	1.0000	− 2.6452	0.0082	0.0041	0.9959
TGARCH vs PGARCH	− 4.1398	0.0000	0.0000	1.0000	− 0.1207	0.9039	0.4520	0.5480	3.8265	0.0001	0.9999	0.0001	1.0876	0.2768	0.8616	0.1384
MGARCH vs PGARCH	− 1.4398	0.1500	0.0750	0.9250	2.3249	0.0201	0.9899	0.0101	5.0187	0.0000	1.0000	0.0000	− 2.0712	0.0401	0.0201	0.9799
Recursive weekly									Rolling window weekly							
GARCH vs EGARCH	− 0.1119	0.9109	0.4555	0.5445	0.8167	0.4144	0.7928	0.2072	1.9166	0.0557	0.9721	0.0279	1.8144	0.0701	0.9649	0.0351
GARCH vs TGARCH	0.4565	0.6482	0.6759	0.3241	0.7167	0.4738	0.7631	0.2369	0.8489	0.3962	0.8019	0.1981	0.4251	0.6709	0.6646	0.3354
GARCH vs MGARCH	0.6868	0.4925	0.7538	0.2462	− 0.9090	0.3637	0.1819	0.8181	1.0858	0.2780	0.8610	0.1390	− 0.6518	0.5148	0.2574	0.7426
GARCH vs PGARCH	− 0.7148	0.4750	0.2375	0.7625	0.5762	0.5647	0.7177	0.2823	1.4296	0.1533	0.9233	0.0767	0.9379	0.3487	0.8257	0.1743
EGARCH vs TGARCH	0.6174	0.5372	0.7314	0.2686	0.0188	0.9850	0.5075	0.4925	− 1.0178	0.3092	0.1546	0.8454	− 1.2000	0.2306	0.1153	0.8847
EGARCH vs MGARCH	0.1460	0.8840	0.5580	0.4420	− 0.8448	0.3986	0.1993	0.8007	− 1.7850	0.0748	0.0374	0.9626	− 1.7448	0.0815	0.0408	0.9592
EGARCH vs PGARCH	− 0.6858	0.4931	0.2465	0.7535	− 0.3421	0.7324	0.3662	0.6338	− 0.6897	0.4906	0.2453	0.7547	− 1.7083	0.0881	0.0440	0.9560
TGARCH vs MGARCH	− 0.4060	0.6849	0.3424	0.6576	− 0.7293	0.4661	0.2330	0.7670	− 0.7593	0.4480	0.2240	0.7760	− 0.4383	0.6614	0.3307	0.6693
TGARCH vs PGARCH	− 1.8396	0.0663	0.0332	0.9668	− 0.6736	0.5008	0.2504	0.7496	1.2211	0.2225	0.8887	0.1113	0.7193	0.4722	0.7639	0.2361
MGARCH vs PGARCH	− 0.7251	0.4686	0.2343	0.7657	0.6012	0.5479	0.7260	0.2740	1.3015	0.1936	0.9032	0.0968	0.9210	0.3574	0.8213	0.1787
Recursive monthly									Rolling window monthly							
GARCH vs EGARCH	− 0.4607	0.6457	0.3228	0.6772	0.2938	0.7693	0.6153	0.3847	0.0560	0.9554	0.5223	0.4777	0.1413	0.8879	0.5561	0.4439
GARCH vs TGARCH	− 1.4661	0.1448	0.0724	0.9276	− 1.5497	0.1234	0.0617	0.9383	− 1.3420	0.1817	0.0908	0.9092	− 0.2086	0.8351	0.4175	0.5825
GARCH vs MGARCH	0.3688	0.7128	0.6436	0.3564	0.3154	0.7529	0.6236	0.3764	0.8793	0.3807	0.8097	0.1903	0.5993	0.5499	0.7250	0.2750
GARCH vs PGARCH	− 5.5805	0.0000	0.0000	1.0000	− 1.6891	0.0933	0.0467	0.9533	− 2.6023	0.0102	0.0051	0.9949	− 0.3151	0.7531	0.3766	0.6234
EGARCH vs TGARCH	− 0.4175	0.6770	0.3385	0.6615	− 0.9016	0.3688	0.1844	0.8156	− 0.5901	0.5561	0.2780	0.7220	− 0.3050	0.7608	0.3804	0.6196
EGARCH vs MGARCH	0.8798	0.3804	0.8098	0.1902	− 0.2286	0.8195	0.4098	0.5902	0.1205	0.9042	0.5479	0.4521	0.0169	0.9865	0.5067	0.4933
EGARCH vs PGARCH	− 5.9298	0.0000	0.0000	1.0000	− 2.4547	0.0153	0.0076	0.9924	− 3.5160	0.0006	0.0003	0.9997	− 0.9427	0.3474	0.1737	0.8263
TGARCH vs MGARCH	1.5346	0.1270	0.9365	0.0635	1.3650	0.1744	0.9128	0.0872	2.4064	0.0174	0.9913	0.0087	1.6194	0.1075	0.9462	0.0538
TGARCH vs PGARCH	− 5.4040	0.0000	0.0000	1.0000	− 1.5732	0.1178	0.0589	0.9411	− 2.4339	0.0161	0.0081	0.9919	− 0.3384	0.7355	0.3678	0.6322
MGARCH vs PGARCH	− 5.8743	0.0000	0.0000	1.0000	− 2.0712	0.0401	0.0201	0.9799	− 3.0209	0.0030	0.0015	0.9985	− 0.6271	0.5316	0.2658	0.7342

1. The columns labelled DM(A) and DM(S) contain t-statistic based on absolute and squared prediction errors, respectively. 2. The null hypothesis of DM-test is that of equal predictive ability of the two models; a significantly positive (negative) t-statistics indicates the benchmark model is dominated by (dominates) the corresponding model

**Table 12:** DM test statistics and *p* values for KLCI Index

Forecast 1 vs Forecast 2	DM(A)	< > *p* value	> *p* value	< *p* value	DM(S)	< > *p* value	> *p* value	< *p* value	DM(A)	< > *p* value	> *p* value	< *p* value	DM(S)	< > *p* value	> *p* value	< *p* value
Recursive daily									Rolling window daily							
GARCH vs EGARCH	1.6920	0.0908	0.9546	0.0454	3.8457	0.0001	0.9999	0.0001	1.0746	0.2827	0.8587	0.1413	3.3640	0.0008	0.9996	0.0004
GARCH vs TGARCH	− 2.5575	0.0106	0.0053	0.9947	− 2.3716	0.0178	0.0089	0.9911	− 3.1592	0.0016	0.0008	0.9992	− 2.3482	0.0189	0.0095	0.9905
GARCH vs MGARCH	5.4386	0.0000	1.0000	0.0000	5.1603	0.0000	1.0000	0.0000	1.6167	0.1061	0.9470	0.0530	1.7572	0.0790	0.9605	0.0395
GARCH vs PGARCH	− 1.0970	0.2727	0.1364	0.8636	0.0569	0.9546	0.5227	0.4773	− 0.1708	0.8644	0.4322	0.5678	1.1112	0.2666	0.8667	0.1333
EGARCH vs TGARCH	− 3.9444	0.0001	0.0000	1.0000	− 4.1053	0.0000	0.0000	1.0000	− 3.6244	0.0003	0.0001	0.9999	− 3.5201	0.0004	0.0002	0.9998
EGARCH vs MGARCH	3.5273	0.0004	0.9998	0.0002	− 1.9035	0.0571	0.0285	0.9715	− 0.0450	0.9641	0.4821	0.5179	− 3.0579	0.0022	0.0011	0.9989
EGARCH vs PGARCH	− 2.3182	0.0205	0.0103	0.9897	− 2.7972	0.0052	0.0026	0.9974	− 0.7727	0.4398	0.2199	0.7801	− 0.7646	0.4446	0.2223	0.7777
TGARCH vs MGARCH	7.1346	0.0000	1.0000	0.0000	4.2056	0.0000	1.0000	0.0000	4.5381	0.0000	1.0000	0.0000	2.7490	0.0060	0.9970	0.0030
TGARCH vs PGARCH	0.6354	0.5252	0.7374	0.2626	1.3333	0.1825	0.9087	0.0913	1.4892	0.1365	0.9317	0.0683	1.9536	0.0508	0.9746	0.0254
MGARCH vs PGARCH	− 3.7378	0.0002	0.0001	0.9999	− 1.1408	0.2540	0.1270	0.8730	− 0.6745	0.5000	0.2500	0.7500	0.8916	0.3727	0.8137	0.1863
Recursive weekly									Rolling window weekly							
GARCH vs EGARCH	− 3.7872	0.0002	0.0001	0.9999	− 1.8320	0.0674	0.0337	0.9663	− 2.6560	0.0081	0.0041	0.9959	− 0.3183	0.7503	0.3752	0.6248
GARCH vs TGARCH	− 4.4596	0.0000	0.0000	1.0000	− 0.8078	0.4195	0.2097	0.7903	− 2.6850	0.0075	0.0037	0.9963	− 1.7576	0.0793	0.0397	0.9603
GARCH vs MGARCH	4.5911	0.0000	1.0000	0.0000	0.9803	0.3273	0.8363	0.1637	2.9218	0.0036	0.9982	0.0018	1.7701	0.0772	0.9614	0.0386
GARCH vs PGARCH	− 4.2314	0.0000	0.0000	1.0000	− 3.8256	0.0001	0.0001	0.9999	− 2.3895	0.0172	0.0086	0.9914	− 1.4218	0.1556	0.0778	0.9222
EGARCH vs TGARCH	2.1113	0.0351	0.9824	0.0176	1.5948	0.1113	0.9444	0.0556	− 0.3884	0.6979	0.3489	0.6511	− 1.3408	0.1805	0.0902	0.9098
EGARCH vs MGARCH	3.8511	0.0001	0.9999	0.0001	1.8619	0.0631	0.9684	0.0316	2.7839	0.0055	0.9972	0.0028	0.3618	0.7176	0.6412	0.3588
EGARCH vs PGARCH	− 3.0717	0.0022	0.0011	0.9989	− 3.5171	0.0005	0.0002	0.9998	− 1.3731	0.1702	0.0851	0.9149	− 1.4200	0.1561	0.0781	0.9219
TGARCH vs MGARCH	4.6261	0.0000	1.0000	0.0000	0.8418	0.4002	0.7999	0.2001	2.7846	0.0055	0.9972	0.0028	1.7961	0.0730	0.9635	0.0365
TGARCH vs PGARCH	− 3.7208	0.0002	0.0001	0.9999	− 3.7552	0.0002	0.0001	0.9999	− 0.7426	0.4580	0.2290	0.7710	− 0.5382	0.5906	0.2953	0.7047
MGARCH vs PGARCH	− 4.2491	0.0000	0.0000	1.0000	− 3.8301	0.0001	0.0001	0.9999	− 2.4554	0.0144	0.0072	0.9928	− 1.4421	0.1498	0.0749	0.9251
Recursive monthly									Rolling window monthly							
GARCH vs EGARCH	0.0779	0.9380	0.5310	0.4690	0.3963	0.6925	0.6538	0.3462	− 0.3002	0.7644	0.3822	0.6178	0.1045	0.9169	0.5415	0.4585
GARCH vs TGARCH	− 1.1408	0.2558	0.1279	0.8721	− 0.4164	0.6777	0.3389	0.6611	− 1.9259	0.0561	0.0280	0.9720	− 0.9190	0.3596	0.1798	0.8202
GARCH vs MGARCH	3.4150	0.0008	0.9996	0.0004	1.0826	0.2808	0.8596	0.1404	NA	NA	NA	NA	NA	NA	NA	NA
GARCH vs PGARCH	− 1.2298	0.2208	0.1104	0.8896	− 1.0321	0.3038	0.1519	0.8481	− 7.6736	0.0000	0.0000	1.0000	− 3.8744	0.0002	0.0001	0.9999
EGARCH vs TGARCH	− 1.2221	0.2237	0.1118	0.8882	− 1.3104	0.1921	0.0961	0.9039	− 2.0996	0.0375	0.0187	0.9813	− 1.6452	0.1021	0.0510	0.9490
EGARCH vs MGARCH	0.0266	0.9788	0.5106	0.4894	− 0.3774	0.7064	0.3532	0.6468	NA	NA	NA	NA	NA	NA	NA	NA
EGARCH vs PGARCH	− 1.2448	0.2152	0.1076	0.8924	− 1.5891	0.1142	0.0571	0.9429	− 8.6373	0.0000	0.0000	1.0000	− 4.3230	0.0000	0.0000	1.0000
TGARCH vs MGARCH	1.2762	0.2039	0.8981	0.1019	0.4347	0.6644	0.6678	0.3322	NA	NA	NA	NA	NA	NA	NA	NA
TGARCH vs PGARCH	− 0.4622	0.6446	0.3223	0.6777	− 1.5585	0.1213	0.0606	0.9394	− 7.3712	0.0000	0.0000	1.0000	− 4.8036	0.0000	0.0000	1.0000
MGARCH vs PGARCH	− 1.3595	0.1761	0.0880	0.9120	− 1.0419	0.2992	0.1496	0.8504	NA	NA	NA	NA	NA	NA	NA	NA

1. The columns labelled DM(A) and DM(S) contain t-statistic based on absolute and squared prediction errors, respectively. 2. The null hypothesis of DM-test is that of equal predictive ability of the two models; a significantly positive (negative) t-statistics indicates the benchmark model is dominated by (dominates) the corresponding model

**Table 13:** DM Test statistics and *p* values for JCI Index

Forecast 1 vs Forecast 2	DM(A)	< > *p* value	> *p* value	< *p* value	DM(S)	< > *p* value	> *p* value	< *p* value	DM(A)	< > *p* value	> *p* value	< *p* value	DM(S)	< > *p* value	> *p* value	< *p* value
Recursive daily									Rolling window daily							
GARCH vs EGARCH	4.6370	0.0000	1.0000	0.0000	2.6396	0.0083	0.9958	0.0042	3.4684	0.0005	0.9997	0.0003	2.8227	0.0048	0.9976	0.0024
GARCH vs TGARCH	0.5832	0.5598	0.7201	0.2799	0.7078	0.4791	0.7604	0.2396	− 0.4036	0.6865	0.3433	0.6567	0.6048	0.5453	0.7273	0.2727
GARCH vs MGARCH	0.9560	0.3392	0.8304	0.1696	0.9949	0.3199	0.8401	0.1599	− 2.2341	0.0256	0.0128	0.9872	− 2.1377	0.0326	0.0163	0.9837
GARCH vs PGARCH	0.2000	0.8415	0.5792	0.4208	1.1065	0.2686	0.8657	0.1343	0.9110	0.3624	0.8188	0.1812	1.0952	0.2735	0.8632	0.1368
EGARCH vs TGARCH	− 3.0237	0.0025	0.0013	0.9987	− 0.6463	0.5181	0.2591	0.7409	− 3.0742	0.0021	0.0011	0.9989	− 0.6270	0.5307	0.2654	0.7346
EGARCH vs MGARCH	− 4.5683	0.0000	0.0000	1.0000	− 2.4357	0.0149	0.0075	0.9925	– 3.7418	0.0002	0.0001	0.9999	− 2.9682	0.0030	0.0015	0.9985
EGARCH vs PGARCH	− 3.8168	0.0001	0.0001	0.9999	− 1.2144	0.2247	0.1124	0.8876	− 2.3661	0.0180	0.0090	0.9910	− 0.5570	0.5775	0.2888	0.7112
TGARCH vs MGARCH	− 0.5485	0.5834	0.2917	0.7083	− 0.6874	0.4919	0.2460	0.7540	0.1339	0.8935	0.5533	0.4467	-0.6670	0.5048	0.2524	0.7476
TGARCH vs PGARCH	− 0.4343	0.6641	0.3320	0.6680	-0.1352	0.8925	0.4462	0.5538	2.2327	0.0256	0.9872	0.0128	0.6630	0.5074	0.7463	0.2537
MGARCH vs PGARCH	0.1428	0.8864	0.5568	0.4432	1.0901	0.2758	0.8621	0.1379	1.1724	0.2411	0.8794	0.1206	1.1589	0.2466	0.8767	0.1233
Recursive weekly									Rolling window weekly							
GARCH vs EGARCH	0.5757	0.5650	0.7175	0.2825	1.1574	0.2476	0.8762	0.1238	0.4608	0.6451	0.6775	0.3225	1.4358	0.1516	0.9242	0.0758
GARCH vs TGARCH	− 3.3100	0.0010	0.0005	0.9995	− 1.1492	0.2509	0.1255	0.8745	− 3.5921	0.0004	0.0002	0.9998	− 0.5874	0.5571	0.2786	0.7214
GARCH vs MGARCH	2.2249	0.0265	0.9868	0.0132	0.9873	0.3239	0.8380	0.1620	− 0.9667	0.3341	0.1671	0.8329	0.1195	0.9049	0.5476	0.4524
GARCH vs PGARCH	− 3.8382	0.0001	0.0001	0.9999	− 1.3643	0.1730	0.0865	0.9135	− 5.0858	0.0000	0.0000	1.0000	− 1.2250	0.2210	0.1105	0.8895
EGARCH vs TGARCH	− 3.8979	0.0001	0.0001	0.9999	− 2.1643	0.0308	0.0154	0.9846	− 4.2839	0.0000	0.0000	1.0000	− 2.7713	0.0058	0.0029	0.9971
EGARCH vs MGARCH	− 0.4096	0.6822	0.3411	0.6589	− 1.0785	0.2812	0.1406	0.8594	− 0.6169	0.5376	0.2688	0.7312	− 1.3753	0.1695	0.0848	0.9152
EGARCH vs PGARCH	− 4.2608	0.0000	0.0000	1.0000	− 2.2708	0.0235	0.0118	0.9882	− 5.3594	0.0000	0.0000	1.0000	− 3.2480	0.0012	0.0006	0.9994
TGARCH vs MGARCH	3.3459	0.0009	0.9996	0.0004	1.1734	0.2411	0.8794	0.1206	3.2812	0.0011	0.9995	0.0005	0.5715	0.5679	0.7161	0.2839
TGARCH vs PGARCH	− 2.0985	0.0363	0.0181	0.9819	− 1.5081	0.1321	0.0660	0.9340	− 2.4205	0.0158	0.0079	0.9921	− 1.7576	0.0793	0.0397	0.9603
MGARCH vs PGARCH	0.5757	0.5650	0.7175	0.2825	1.1574	0.2476	0.8762	0.1238	− 4.7785	0.0000	0.0000	1.0000	− 1.1632	0.2452	0.1226	0.8774
Recursive monthly									Rolling window monthly							
GARCH vs EGARCH	− 3.9896	0.0001	0.0001	0.9999	− 2.9315	0.0039	0.0020	0.9980	0.5628	0.5744	0.7128	0.2872	1.3262	0.1869	0.9065	0.0935
GARCH vs TGARCH	− 3.8280	0.0002	0.0001	0.9999	− 2.5648	0.0113	0.0057	0.9943	− 1.5331	0.1274	0.0637	0.9363	− 1.0423	0.2990	0.1495	0.8505
GARCH vs MGARCH	1.5076	0.1338	0.9331	0.0669	− 0.2530	0.8006	0.4003	0.5997	− 4.0777	0.0001	0.0000	1.0000	− 3.7775	0.0002	0.0001	0.9999
GARCH vs PGARCH	− 2.2652	0.0250	0.0125	0.9875	− 2.1852	0.0305	0.0152	0.9848	− 2.0589	0.0413	0.0206	0.9794	− 2.0946	0.0379	0.0190	0.9810
EGARCH vs TGARCH	2.0662	0.0406	0.9797	0.0203	1.5945	0.1130	0.9435	0.0565	− 1.2940	0.1978	0.0989	0.9011	− 1.8435	0.0674	0.0337	0.9663
EGARCH vs MGARCH	4.0244	0.0001	1.0000	0.0000	2.9349	0.0039	0.9981	0.0019	− 0.9810	0.3283	0.1642	0.8358	− 1.4297	0.1550	0.0775	0.9225
EGARCH vs PGARCH	1.4036	0.1626	0.9187	0.0813	− 0.4481	0.6548	0.3274	0.6726	− 1.4909	0.1382	0.0691	0.9309	− 1.9045	0.0589	0.0294	0.9706
TGARCH vs MGARCH	3.9616	0.0001	0.9999	0.0001	2.6077	0.0101	0.9950	0.0050	1.1712	0.2434	0.8783	0.1217	0.9461	0.3457	0.8272	0.1728
TGARCH vs PGARCH	− 0.5048	0.6144	0.3072	0.6928	− 1.9459	0.0536	0.0268	0.9732	− 1.8788	0.0623	0.0311	0.9689	− 2.2280	0.0274	0.0137	0.9863
MGARCH vs PGARCH	− 2.3181	0.0218	0.0109	0.9891	− 2.1936	0.0298	0.0149	0.9851	− 1.8281	0.0696	0.0348	0.9652	− 2.0594	0.0412	0.0206	0.9794

1. The columns labelled DM(A) and DM(S) contain t-statistic based on absolute and squared prediction errors, respectively. 2. The null hypothesis of DM-test is that of equal predictive ability of the two models; a significantly positive (negative) t-statistics indicates the benchmark model is dominated by (dominates) the corresponding model

**Table 14:** DM Test statistics and *p* values for SET Index

Forecast 1 vs Forecast 2	DM(A)	< > *p* value	> *p* value	< *p* value	DM(S)	< > *p* value	> *p* value	< *p* value	DM(A)	< > *p* value	> *p* value	< *p* value	DM(S)	< > *p* value	> *p* value	< *p* value
Recursive daily									Rolling window daily							
GARCH vs EGARCH	1.8401	0.0659	0.9671	0.0329	0.2096	0.8340	0.5830	0.4170	1.3697	0.1709	0.9146	0.0854	0.0151	0.9880	0.5060	0.4940
GARCH vs TGARCH	1.1774	0.2391	0.8804	0.1196	0.7933	0.4277	0.7862	0.2138	− 0.2330	0.8158	0.4079	0.5921	0.6644	0.5065	0.7468	0.2532
GARCH vs MGARCH	3.2638	0.0011	0.9994	0.0006	2.2313	0.0257	0.9871	0.0129	1.8260	0.0680	0.9660	0.0340	− 0.2714	0.7861	0.3930	0.6070
GARCH vs PGARCH	− 2.2789	0.0227	0.0114	0.9886	− 0.3759	0.7070	0.3535	0.6465	− 0.9638	0.3352	0.1676	0.8324	− 0.2701	0.7871	0.3936	0.6064
EGARCH vs TGARCH	− 1.5811	0.1140	0.0570	0.9430	− 0.0031	0.9975	0.4988	0.5012	− 1.6009	0.1095	0.0548	0.9452	0.1804	0.8569	0.5716	0.4284
EGARCH vs MGARCH	− 1.2593	0.2080	0.1040	0.8960	− 0.1820	0.8556	0.4278	0.5722	− 1.0930	0.2745	0.1373	0.8627	− 0.0201	0.9840	0.4920	0.5080
EGARCH vs PGARCH	− 4.1522	0.0000	0.0000	1.0000	− 0.7027	0.4823	0.2411	0.7589	− 2.5849	0.0098	0.0049	0.9951	− 0.2655	0.7907	0.3953	0.6047
TGARCH vs MGARCH	0.6023	0.5470	0.7265	0.2735	− 0.6802	0.4964	0.2482	0.7518	1.1132	0.2657	0.8671	0.1329	− 0.6713	0.5021	0.2510	0.7490
TGARCH vs PGARCH	− 3.6526	0.0003	0.0001	0.9999	− 1.2850	0.1989	0.0994	0.9006	− 1.2258	0.2204	0.1102	0.8898	− 0.9572	0.3386	0.1693	0.8307
MGARCH vs PGARCH	− 3.2538	0.0012	0.0006	0.9994	− 0.4282	0.6685	0.3343	0.6657	− 1.4867	0.1372	0.0686	0.9314	− 0.2597	0.7951	0.3976	0.6024
Recursive weekly									Rolling window weekly							
GARCH vs EGARCH	1.7184	0.0862	0.9569	0.0431	1.8496	0.0649	0.9676	0.0324	3.1809	0.0015	0.9992	0.0008	2.2923	0.0222	0.9889	0.0111
GARCH vs TGARCH	− 3.5077	0.0005	0.0002	0.9998	− 2.2648	0.0239	0.0119	0.9881	− 5.0915	0.0000	0.0000	1.0000	− 3.1833	0.0015	0.0008	0.9992
GARCH vs MGARCH	1.3468	0.1786	0.9107	0.0893	0.5541	0.5797	0.7101	0.2899	1.0870	0.2775	0.8613	0.1387	1.4277	0.1539	0.9231	0.0769
GARCH vs PGARCH	3.5073	0.0005	0.9998	0.0002	2.1377	0.0329	0.9835	0.0165	1.8654	0.0626	0.9687	0.0313	0.6397	0.5226	0.7387	0.2613
EGARCH vs TGARCH	− 3.0162	0.0027	0.0013	0.9987	− 2.6556	0.0081	0.0041	0.9959	− 4.8865	0.0000	0.0000	1.0000	− 2.9168	0.0037	0.0018	0.9982
EGARCH vs MGARCH	− 1.7051	0.0887	0.0443	0.9557	− 1.8589	0.0635	0.0318	0.9682	− 3.2595	0.0012	0.0006	0.9994	− 2.3443	0.0194	0.0097	0.9903
EGARCH vs PGARCH	2.3682	0.0182	0.9909	0.0091	2.2113	0.0274	0.9863	0.0137	− 2.2572	0.0244	0.0122	0.9878	− 0.9131	0.3615	0.1808	0.8192
TGARCH vs MGARCH	3.5124	0.0005	0.9998	0.0002	2.2448	0.0251	0.9874	0.0126	4.8982	0.0000	1.0000	0.0000	3.0405	0.0025	0.9988	0.0012
TGARCH vs PGARCH	3.9982	0.0001	1.0000	0.0000	2.8171	0.0050	0.9975	0.0025	4.0357	0.0001	1.0000	0.0000	1.9582	0.0507	0.9747	0.0253
MGARCH vs PGARCH	3.5129	0.0005	0.9998	0.0002	2.1495	0.0320	0.9840	0.0160	1.8352	0.0670	0.9665	0.0335	0.4979	0.6188	0.6906	0.3094
Recursive monthly									Rolling window monthly							
GARCH vs EGARCH	− 3.0459	0.0028	0.0014	0.9986	− 0.7511	0.4538	0.2269	0.7731	− 1.4844	0.1399	0.0699	0.9301	1.1940	0.2344	0.8828	0.1172
GARCH vs TGARCH	− 2.0352	0.0436	0.0218	0.9782	− 1.4371	0.1528	0.0764	0.9236	− 1.3491	0.1794	0.0897	0.9103	− 1.3875	0.1674	0.0837	0.9163
GARCH vs MGARCH	− 2.6308	0.0094	0.0047	0.9953	− 0.1857	0.8529	0.4265	0.5735	− 0.7057	0.4815	0.2407	0.7593	1.1633	0.2466	0.8767	0.1233
GARCH vs PGARCH	0.1651	0.8691	0.5655	0.4345	− 0.3418	0.7330	0.3665	0.6335	1.7418	0.0837	0.9582	0.0418	1.3631	0.1750	0.9125	0.0875
EGARCH vs TGARCH	2.2479	0.0261	0.9870	0.0130	− 0.2145	0.8305	0.4152	0.5848	1.0953	0.2752	0.8624	0.1376	− 1.2868	0.2002	0.1001	0.8999
EGARCH vs MGARCH	2.8724	0.0047	0.9977	0.0023	0.7171	0.4745	0.7628	0.2372	1.4497	0.1493	0.9254	0.0746	− 1.1209	0.2642	0.1321	0.8679
EGARCH vs PGARCH	2.7235	0.0073	0.9964	0.0036	0.0248	0.9802	0.5099	0.4901	3.8434	0.0002	0.9999	0.0001	1.2211	0.2240	0.8880	0.1120
TGARCH vs MGARCH	1.4309	0.1546	0.9227	0.0773	1.4390	0.1523	0.9239	0.0761	0.5003	0.6176	0.6912	0.3088	1.5056	0.1343	0.9328	0.0672
TGARCH vs PGARCH	0.9966	0.3206	0.8397	0.1603	0.2389	0.8115	0.5942	0.4058	1.7561	0.0812	0.9594	0.0406	1.3801	0.1697	0.9152	0.0848
MGARCH vs PGARCH	0.4163	0.6778	0.6611	0.3389	− 0.3222	0.7478	0.3739	0.6261	1.9483	0.0533	0.9733	0.0267	1.3081	0.1929	0.9036	0.0964

1. The columns labelled DM(A) and DM(S) contain t-statistic based on absolute and squared prediction errors, respectively. 2. The null hypothesis of DM-test is that of equal predictive ability of the two models; a significantly positive (negative) *t* statistics indicates the benchmark model is dominated by (dominates) the corresponding model

**Table 15:** DM test statistics and *p* values for SSE Index

Forecast 1 vs Forecast 2	DM(A)	< > *p* value	> *p* value	< *p* value	DM(S)	< > *p* value	> *p* value	< *p* value	DM(A)	< > *p* value	> *p* value	< *p* value	DM(S)	< > *p* value	> *p* value	< *p* value
Recursive daily									Rolling window daily							
GARCH vs EGARCH	2.0171	0.0438	0.9781	0.0219	1.4789	0.1393	0.9304	0.0696	3.8382	0.0001	0.9999	0.0001	0.7217	0.4705	0.7647	0.2353
GARCH vs TGARCH	− 0.6216	0.5343	0.2671	0.7329	0.4923	0.6225	0.6887	0.3113	2.1992	0.0279	0.9860	0.0140	− 0.6527	0.5140	0.2570	0.7430
GARCH vs MGARCH	3.8078	0.0001	0.9999	0.0001	2.9873	0.0028	0.9986	0.0014	− 1.9501	0.0513	0.0256	0.9744	0.5668	0.5709	0.7146	0.2854
GARCH vs PGARCH	− 1.5902	0.1119	0.0560	0.9440	− 0.7477	0.4547	0.2274	0.7726	− 0.6951	0.4870	0.2435	0.7565	− 0.6174	0.5370	0.2685	0.7315
EGARCH vs TGARCH	− 3.1050	0.0019	0.0010	0.9990	− 0.8878	0.3747	0.1874	0.8126	− 3.7318	0.0002	0.0001	0.9999	− 0.7939	0.4273	0.2137	0.7863
EGARCH vs MGARCH	0.7751	0.4384	0.7808	0.2192	− 0.3977	0.6909	0.3454	0.6546	− 3.8602	0.0001	0.0001	0.9999	− 0.7081	0.4789	0.2395	0.7605
EGARCH vs PGARCH	− 7.4058	0.0000	0.0000	1.0000	− 4.1153	0.0000	0.0000	1.0000	− 8.8025	0.0000	0.0000	1.0000	− 2.3312	0.0198	0.0099	0.9901
TGARCH vs MGARCH	5.6427	0.0000	1.0000	0.0000	0.7656	0.4440	0.7780	0.2220	− 2.2387	0.0253	0.0126	0.9874	0.6550	0.5125	0.7437	0.2563
TGARCH vs PGARCH	− 1.3623	0.1732	0.0866	0.9134	− 1.2095	0.2266	0.1133	0.8867	− 0.8841	0.3767	0.1884	0.8116	− 0.5559	0.5783	0.2892	0.7108
MGARCH vs PGARCH	− 5.1290	0.0000	0.0000	1.0000	− 1.7660	0.0775	0.0388	0.9612	− 0.6635	0.5071	0.2535	0.7465	− 0.6305	0.5284	0.2642	0.7358
Recursive weekly									Rolling window weekly							
GARCH vs EGARCH	3.2787	0.0011	0.9994	0.0006	2.2949	0.0221	0.9890	0.0110	3.7391	0.0002	0.9999	0.0001	1.7114	0.0875	0.9562	0.0438
GARCH vs TGARCH	− 1.9441	0.0524	0.0262	0.9738	− 1.1746	0.2406	0.1203	0.8797	2.7886	0.0055	0.9973	0.0027	0.5899	0.5555	0.7223	0.2777
GARCH vs MGARCH	− 2.2902	0.0224	0.0112	0.9888	− 1.1805	0.2383	0.1191	0.8809	0.0909	0.9276	0.5362	0.4638	0.8265	0.4089	0.7956	0.2044
GARCH vs PGARCH	− 3.5254	0.0005	0.0002	0.9998	− 2.4534	0.0144	0.0072	0.9928	0.4385	0.6612	0.6694	0.3306	− 0.8229	0.4109	0.2054	0.7946
EGARCH vs TGARCH	− 3.9137	0.0001	0.0001	0.9999	− 2.4048	0.0165	0.0082	0.9918	− 3.1482	0.0017	0.0009	0.9991	− 1.9540	0.0512	0.0256	0.9744
EGARCH vs MGARCH	− 3.8074	0.0002	0.0001	0.9999	− 2.3921	0.0171	0.0085	0.9915	− 3.4459	0.0006	0.0003	0.9997	− 1.1785	0.2391	0.1195	0.8805
EGARCH vs PGARCH	− 3.8389	0.0001	0.0001	0.9999	− 2.5957	0.0097	0.0048	0.9952	− 2.9484	0.0033	0.0017	0.9983	− 1.6622	0.0970	0.0485	0.9515
TGARCH vs MGARCH	0.2828	0.7775	0.6113	0.3887	0.5203	0.6030	0.6985	0.3015	− 2.3065	0.0214	0.0107	0.9893	0.2070	0.8361	0.5820	0.4180
TGARCH vs PGARCH	− 3.3522	0.0009	0.0004	0.9996	− 2.5591	0.0107	0.0054	0.9946	− 1.2916	0.1970	0.0985	0.9015	− 0.8829	0.3777	0.1888	0.8112
MGARCH vs PGARCH	− 3.2364	0.0013	0.0006	0.9994	− 2.4623	0.0141	0.0070	0.9930	0.3185	0.7502	0.6249	0.3751	− 0.9746	0.3302	0.1651	0.8349
Recursive monthly									Rolling window monthly							
GARCH vs EGARCH	0.8134	0.4173	0.7913	0.2087	0.9893	0.3242	0.8379	0.1621	− 0.2385	0.8118	0.4059	0.5941	− 0.1030	0.9181	0.4591	0.5409
GARCH vs TGARCH	− 1.7492	0.0823	0.0412	0.9588	− 1.7283	0.0861	0.0430	0.9570	1.5589	0.1212	0.9394	0.0606	− 0.0598	0.9524	0.4762	0.5238
GARCH vs MGARCH	1.4013	0.1633	0.9184	0.0816	− 0.0312	0.9752	0.4876	0.5124	− 1.0000	0.3190	0.1595	0.8405	− 1.0000	0.3190	0.1595	0.8405
GARCH vs PGARCH	− 2.0286	0.0443	0.0222	0.9778	− 2.4331	0.0162	0.0081	0.9919	1.5773	0.1169	0.9416	0.0584	1.1237	0.2630	0.8685	0.1315
EGARCH vs TGARCH	− 2.7957	0.0059	0.0029	0.9971	− 3.1673	0.0019	0.0009	0.9991	1.4634	0.1455	0.9272	0.0728	0.6148	0.5397	0.7302	0.2698
EGARCH vs MGARCH	0.4754	0.6352	0.6824	0.3176	− 0.8618	0.3902	0.1951	0.8049	− 1.0000	0.3190	0.1595	0.8405	− 1.0000	0.3190	0.1595	0.8405
EGARCH vs PGARCH	− 2.2892	0.0235	0.0118	0.9882	− 2.7546	0.0066	0.0033	0.9967	1.4564	0.1474	0.9263	0.0737	1.1459	0.2537	0.8731	0.1269
TGARCH vs MGARCH	2.4147	0.0170	0.9915	0.0085	1.6522	0.1006	0.9497	0.0503	− 1.0000	0.3190	0.1595	0.8405	− 1.0000	0.3190	0.1595	0.8405
TGARCH vs PGARCH	− 0.9356	0.3510	0.1755	0.8245	− 1.7381	0.0843	0.0422	0.9578	0.1711	0.8644	0.5678	0.4322	1.1141	0.2671	0.8665	0.1335
MGARCH vs PGARCH	− 2.5218	0.0127	0.0064	0.9936	− 2.2786	0.0241	0.0121	0.9879	1.0000	0.3190	0.8405	0.1595	1.0000	0.3190	0.8405	0.1595

1. The columns labelled DM(A) and DM(S) contain t-statistic based on absolute and squared prediction errors, respectively. 2. The null hypothesis of DM-test is that of equal predictive ability of the two models; a significantly positive (negative) *t* statistics indicates the benchmark model is dominated by (dominates) the corresponding model

**Table 16:** DM test statistics and *p* values for TAIEX Index

Forecast 1 vs Forecast 2	DM(A)	< > *p* value	> *p* value	< *p* value	DM(S)	< > *p* value	> *p* value	< *p* value	DM(A)	< > *p* value	> *p* value	< *p* value	DM(S)	< > *p* value	> *p* value	< *p* value
Recursive daily									Rolling window daily							
GARCH vs EGARCH	0.9645	0.3349	0.8326	0.1674	1.2098	0.2264	0.8868	0.1132	1.1910	0.2338	0.8831	0.1169	0.4381	0.6614	0.6693	0.3307
GARCH vs TGARCH	− 4.7049	0.0000	0.0000	1.0000	0.2317	0.8168	0.5916	0.4084	− 4.7427	0.0000	0.0000	1.0000	0.2323	0.8164	0.5918	0.4082
GARCH vs MGARCH	− 0.0351	0.9720	0.4860	0.5140	1.6573	0.0976	0.9512	0.0488	2.6383	0.0084	0.9958	0.0042	1.7935	0.0730	0.9635	0.0365
GARCH vs PGARCH	− 4.2499	0.0000	0.0000	1.0000	− 1.5768	0.1149	0.0575	0.9425	− 3.3243	0.0009	0.0004	0.9996	− 0.9642	0.3350	0.1675	0.8325
EGARCH vs TGARCH	− 6.1942	0.0000	0.0000	1.0000	− 1.4699	0.1417	0.0708	0.9292	− 6.3921	0.0000	0.0000	1.0000	− 0.2688	0.7881	0.3941	0.6059
EGARCH vs MGARCH	− 0.9759	0.3292	0.1646	0.8354	− 1.1807	0.2378	0.1189	0.8811	− 1.1499	0.2503	0.1251	0.8749	− 0.4080	0.6833	0.3417	0.6583
EGARCH vs PGARCH	− 5.8275	0.0000	0.0000	1.0000	− 4.5383	0.0000	0.0000	1.0000	− 4.8912	0.0000	0.0000	1.0000	− 1.3343	0.1822	0.0911	0.9089
TGARCH vs MGARCH	4.7689	0.0000	1.0000	0.0000	− 0.1816	0.8559	0.4280	0.5720	4.8260	0.0000	1.0000	0.0000	− 0.1938	0.8463	0.4232	0.5768
TGARCH vs PGARCH	− 1.5109	0.1309	0.0655	0.9345	− 2.6643	0.0078	0.0039	0.9961	− 0.3739	0.7085	0.3542	0.6458	− 1.6736	0.0943	0.0472	0.9528
MGARCH vs PGARCH	− 4.2731	0.0000	0.0000	1.0000	− 1.6390	0.1013	0.0507	0.9493	− 3.3668	0.0008	0.0004	0.9996	− 1.0045	0.3152	0.1576	0.8424
Recursive weekly									Rolling window weekly							
GARCH vs EGARCH	− 2.0911	0.0369	0.0185	0.9815	− 0.8522	0.3944	0.1972	0.8028	− 0.2957	0.7676	0.3838	0.6162	0.1417	0.8874	0.5563	0.4437
GARCH vs TGARCH	− 0.7275	0.4672	0.2336	0.7664	1.3618	0.1738	0.9131	0.0869	0.4796	0.6317	0.6842	0.3158	1.1983	0.2313	0.8844	0.1156
GARCH vs MGARCH	− 3.1372	0.0018	0.0009	0.9991	− 1.9617	0.0503	0.0251	0.9749	− 1.4777	0.1400	0.0700	0.9300	− 0.9252	0.3552	0.1776	0.8224
GARCH vs PGARCH	− 1.8639	0.0628	0.0314	0.9686	− 2.0191	0.0439	0.0220	0.9780	− 2.5127	0.0122	0.0061	0.9939	− 1.9215	0.0551	0.0276	0.9724
EGARCH vs TGARCH	1.2962	0.1954	0.9023	0.0977	2.1000	0.0361	0.9819	0.0181	0.7093	0.4784	0.7608	0.2392	1.0279	0.3044	0.8478	0.1522
EGARCH vs MGARCH	1.6081	0.1083	0.9458	0.0542	0.4465	0.6554	0.6723	0.3277	0.0136	0.9891	0.5054	0.4946	− 0.3452	0.7300	0.3650	0.6350
EGARCH vs PGARCH	− 0.6826	0.4951	0.2476	0.7524	− 1.8506	0.0647	0.0324	0.9676	− 2.7285	0.0065	0.0033	0.9967	− 2.0978	0.0363	0.0182	0.9818
TGARCH vs MGARCH	0.2598	0.7951	0.6024	0.3976	− 1.4781	0.1399	0.0699	0.9301	− 0.6864	0.4927	0.2464	0.7536	− 1.2048	0.2287	0.1144	0.8856
TGARCH vs PGARCH	− 1.2256	0.2208	0.1104	0.8896	− 2.0462	0.0412	0.0206	0.9794	− 2.4601	0.0142	0.0071	0.9929	− 1.9862	0.0475	0.0237	0.9763
MGARCH vs PGARCH	− 1.6316	0.1033	0.0516	0.9484	− 1.9564	0.0509	0.0254	0.9746	− 2.4393	0.0150	0.0075	0.9925	− 1.9212	0.0552	0.0276	0.9724
Recursive monthly									Rolling window monthly							
GARCH vs EGARCH	− 9.7431	0.0000	0.0000	1.0000	− 6.8476	0.0000	0.0000	1.0000	− 3.4547	0.0007	0.0004	0.9996	− 1.4641	0.1453	0.0727	0.9273
GARCH vs TGARCH	− 2.5822	0.0108	0.0054	0.9946	− 0.3941	0.6941	0.3470	0.6530	− 0.7993	0.4254	0.2127	0.7873	0.2942	0.7691	0.6155	0.3845
GARCH vs MGARCH	NA	NA	NA	NA	NA	NA	NA	NA	NA	NA	NA	NA	NA	NA	NA	NA
GARCH vs PGARCH	− 3.2389	0.0015	0.0007	0.9993	− 1.8523	0.0660	0.0330	0.9670	0.6402	0.5231	0.7385	0.2615	0.6768	0.4997	0.7502	0.2498
EGARCH vs TGARCH	8.9016	0.0000	1.0000	0.0000	4.9588	0.0000	1.0000	0.0000	3.0196	0.0030	0.9985	0.0015	2.2357	0.0269	0.9865	0.0135
EGARCH vs MGARCH	NA	NA	NA	NA	NA	NA	NA	NA	NA	NA	NA	NA	NA	NA	NA	NA
EGARCH vs PGARCH	9.2551	0.0000	1.0000	0.0000	4.6488	0.0000	1.0000	0.0000	4.2142	0.0000	1.0000	0.0000	2.6629	0.0087	0.9957	0.0043
TGARCH vs MGARCH	NA	NA	NA	NA	NA	NA	NA	NA	NA	NA	NA	NA	NA	NA	NA	NA
TGARCH vs PGARCH	− 1.4070	0.1616	0.0808	0.9192	− 1.2948	0.1974	0.0987	0.9013	1.6472	0.1018	0.9491	0.0509	0.6958	0.4877	0.7561	0.2439
MGARCH vs PGARCH	NA	NA	NA	NA	NA	NA	NA	NA	NA	NA	NA	NA	NA	NA	NA	NA

1. The columns labelled DM(A) and DM(S) contain t-statistic based on absolute and squared prediction errors, respectively. 2. The null hypothesis of DM-test is that of equal predictive ability of the two models; a significantly positive (negative) *t* statistics indicates the benchmark model is dominated by (dominates) the corresponding model

**Table 17:** DM test statistics and *p *values for KOSPI Index

Forecast 1 vs Forecast 2	DM(A)	< > *p *value	> *p *value	< *p *value	DM(S)	< > *p *value	> *p *value	< *p *value	DM(A)	< > *p *value	> *p *value	< *p *value	DM(S)	< > *p *value	> *p *value	< *p *value
Recursive daily									Rolling window daily							
GARCH vs EGARCH	2.5370	0.0112	0.9944	0.0056	2.6951	0.0071	0.9965	0.0035	0.8975	0.3695	0.8152	0.1848	2.8137	0.0049	0.9975	0.0025
GARCH vs TGARCH	− 0.4477	0.6544	0.3272	0.6728	1.0465	0.2954	0.8523	0.1477	− 2.9034	0.0037	0.0019	0.9981	0.9763	0.3290	0.8355	0.1645
GARCH vs MGARCH	6.9437	0.0000	1.0000	0.0000	2.5426	0.0111	0.9945	0.0055	− 0.9051	0.3655	0.1828	0.8172	− 1.5928	0.1113	0.0557	0.9443
GARCH vs PGARCH	− 3.1087	0.0019	0.0009	0.9991	0.3646	0.7155	0.6423	0.3577	− 4.2694	0.0000	0.0000	1.0000	0.4151	0.6781	0.6609	0.3391
EGARCH vs TGARCH	− 6.1942	0.0000	0.0000	1.0000	− 1.4699	0.1417	0.0708	0.9292	− 3.2004	0.0014	0.0007	0.9993	− 1.1363	0.2559	0.1280	0.8720
EGARCH vs MGARCH	− 2.8100	0.0050	0.0025	0.9975	− 0.7476	0.4548	0.2274	0.7726	− 0.9122	0.3618	0.1809	0.8191	− 2.7897	0.0053	0.0027	0.9973
EGARCH vs PGARCH	− 4.6720	0.0000	0.0000	1.0000	− 2.2567	0.0241	0.0120	0.9880	− 5.1593	0.0000	0.0000	1.0000	− 3.2145	0.0013	0.0007	0.9993
TGARCH vs MGARCH	0.5306	0.5958	0.7021	0.2979	− 1.0405	0.2982	0.1491	0.8509	2.8164	0.0049	0.9976	0.0024	− 1.0038	0.3156	0.1578	0.8422
TGARCH vs PGARCH	− 3.1346	0.0017	0.0009	0.9991	− 2.2352	0.0255	0.0127	0.9873	− 2.7679	0.0057	0.0028	0.9972	− 1.2428	0.2141	0.1070	0.8930
MGARCH vs PGARCH	− 3.1497	0.0017	0.0008	0.9992	0.3575	0.7207	0.6396	0.3604	− 4.2243	0.0000	0.0000	1.0000	0.4808	0.6307	0.6847	0.3153
Recursive weekly									Rolling window weekly							
GARCH vs EGARCH	1.2952	0.1958	0.9021	0.0979	− 0.3591	0.7196	0.3598	0.6402	0.9247	0.3555	0.8223	0.1777	2.0549	0.0403	0.9798	0.0202
GARCH vs TGARCH	− 1.9267	0.0545	0.0272	0.9728	1.0130	0.3114	0.8443	0.1557	− 3.2808	0.0011	0.0005	0.9995	0.8787	0.3799	0.8100	0.1900
GARCH vs MGARCH	5.6476	0.0000	1.0000	0.0000	2.4140	0.0161	0.9920	0.0080	2.6984	0.0072	0.9964	0.0036	− 1.2630	0.2071	0.1035	0.8965
GARCH vs PGARCH	3.0623	0.0023	0.9989	0.0011	2.2790	0.0230	0.9885	0.0115	− 2.2789	0.0230	0.0115	0.9885	2.4720	0.0137	0.9931	0.0069
EGARCH vs TGARCH	− 1.5052	0.1328	0.0664	0.9336	0.4789	0.6321	0.6839	0.3161	− 2.4766	0.0135	0.0068	0.9932	− 0.8198	0.4127	0.2063	0.7937
EGARCH vs MGARCH	− 1.1622	0.2456	0.1228	0.8772	0.3859	0.6997	0.6502	0.3498	− 0.7943	0.4273	0.2137	0.7863	− 2.1124	0.0351	0.0175	0.9825
EGARCH vs PGARCH	− 0.8259	0.4092	0.2046	0.7954	0.5661	0.5716	0.7142	0.2858	− 4.1083	0.0000	0.0000	1.0000	− 1.2130	0.2256	0.1128	0.8872
TGARCH vs MGARCH	2.6595	0.0080	0.9960	0.0040	− 0.8986	0.3692	0.1846	0.8154	3.4569	0.0006	0.9997	0.0003	− 0.8951	0.3711	0.1855	0.8145
TGARCH vs PGARCH	5.1003	0.0000	1.0000	0.0000	0.3783	0.7054	0.6473	0.3527	0.6380	0.5237	0.7382	0.2618	0.5014	0.6163	0.6919	0.3081
MGARCH vs PGARCH	2.2885	0.0224	0.9888	0.0112	2.1930	0.0287	0.9857	0.0143	− 2.4646	0.0140	0.0070	0.9930	2.4828	0.0133	0.9933	0.0067
Recursive monthly									Rolling window monthly							
GARCH vs EGARCH	0.4723	0.6374	0.6813	0.3187	− 0.0046	0.9964	0.4982	0.5018	− 2.4031	0.0175	0.0088	0.9912	− 1.2907	0.1989	0.0994	0.9006
GARCH vs TGARCH	− 1.7375	0.0844	0.0422	0.9578	− 1.3154	0.1905	0.0952	0.9048	− 1.8626	0.0645	0.0323	0.9677	− 1.5522	0.1228	0.0614	0.9386
GARCH vs MGARCH	3.6192	0.0004	0.9998	0.0002	2.9323	0.0039	0.9980	0.0020	− 3.8199	0.0002	0.0001	0.9999	− 2.5659	0.0113	0.0056	0.9944
GARCH vs PGARCH	2.5568	0.0116	0.9942	0.0058	1.1458	0.2537	0.8731	0.1269	− 0.9119	0.3633	0.1817	0.8183	− 0.3895	0.6975	0.3487	0.6513
EGARCH vs TGARCH	− 3.6686	0.0003	0.0002	0.9998	− 2.8657	0.0048	0.0024	0.9976	0.7658	0.4450	0.7775	0.2225	− 1.1962	0.2336	0.1168	0.8832
EGARCH vs MGARCH	− 0.2632	0.7928	0.3964	0.6036	0.0674	0.9463	0.5268	0.4732	− 3.8016	0.0002	0.0001	0.9999	− 2.5656	0.0113	0.0057	0.9943
EGARCH vs PGARCH	2.7139	0.0074	0.9963	0.0037	1.4881	0.1389	0.9306	0.0694	1.1774	0.2410	0.8795	0.1205	0.8805	0.3801	0.8100	0.1900
TGARCH vs MGARCH	1.8663	0.0640	0.9680	0.0320	1.3517	0.1786	0.9107	0.0893	− 3.8025	0.0002	0.0001	0.9999	− 2.5655	0.0113	0.0057	0.9943
TGARCH vs PGARCH	3.3799	0.0009	0.9995	0.0005	1.9216	0.0566	0.9717	0.0283	0.6060	0.5455	0.7273	0.2727	1.1299	0.2604	0.8698	0.1302
MGARCH vs PGARCH	2.3964	0.0178	0.9911	0.0089	2.1930	0.0287	0.9857	0.0143	3.8178	0.0002	0.9999	0.0001	2.5662	0.0113	0.9944	0.0056

1. The columns labelled DM(A) and DM(S) contain t-statistic based on absolute and squared prediction errors, respectively. 2. The null hypothesis of DM-test is that of equal predictive ability of the two models; a significantly positive (negative) *t* statistics indicates the benchmark model is dominated by (dominates) the corresponding model

**Table 18:** DM test statistics and *p* values for PSE Index

Forecast 1 vs Forecast 2	DM(A)	< > *p* value	> *p* value	< *p* value	DM(S)	< > *p* value	> *p* value	< *p* value	DM(A)	< > *p* value	> *p* value	< *p* value	DM(S)	< > *p* value	> *p* value	< *p* value
Recursive daily									Rolling window daily							
GARCH vs EGARCH	3.6207	0.0003	0.9999	0.0001	2.8060	0.0051	0.9975	0.0025	2.8631	0.0042	0.9979	0.0021	3.5480	0.0004	0.9998	0.0002
GARCH vs TGARCH	− 1.6004	0.1096	0.0548	0.9452	0.5024	0.6155	0.6923	0.3077	− 0.7051	0.4808	0.2404	0.7596	0.8094	0.4184	0.7908	0.2092
GARCH vs MGARCH	− 1.8889	0.0590	0.0295	0.9705	0.2083	0.8350	0.5825	0.4175	0.6201	0.5352	0.7324	0.2676	1.2869	0.1982	0.9009	0.0991
GARCH vs PGARCH	− 0.3958	0.6923	0.3461	0.6539	1.6810	0.0929	0.9536	0.0464	0.4984	0.6182	0.6909	0.3091	2.0794	0.0377	0.9812	0.0188
EGARCH vs TGARCH	− 3.8710	0.0001	0.0001	0.9999	− 1.4905	0.1362	0.0681	0.9319	− 2.8523	0.0044	0.0022	0.9978	− 1.6715	0.0947	0.0474	0.9526
EGARCH vs MGARCH	− 4.2562	0.0000	0.0000	1.0000	− 2.8009	0.0051	0.0026	0.9974	− 2.6773	0.0075	0.0037	0.9963	− 3.4003	0.0007	0.0003	0.9997
EGARCH vs PGARCH	− 3.7271	0.0002	0.0001	0.9999	− 1.0254	0.3053	0.1526	0.8474	− 2.7877	0.0053	0.0027	0.9973	− 1.1960	0.2318	0.1159	0.8841
TGARCH vs MGARCH	0.5618	0.5743	0.7129	0.2871	− 0.5481	0.5837	0.2918	0.7082	0.9190	0.3582	0.8209	0.1791	− 0.7114	0.4769	0.2384	0.7616
TGARCH vs PGARCH	1.1472	0.2514	0.8743	0.1257	1.8135	0.0699	0.9651	0.0349	1.4103	0.1586	0.9207	0.0793	1.9219	0.0547	0.9726	0.0274
MGARCH vs PGARCH	0.5848	0.5588	0.7206	0.2794	1.9503	0.0512	0.9744	0.0256	0.3003	0.7640	0.6180	0.3820	2.1237	0.0338	0.9831	0.0169
Recursive weekly									Rolling window weekly							
GARCH vs EGARCH	3.9926	0.0001	1.0000	0.0000	1.8566	0.0638	0.9681	0.0319	2.8893	0.0040	0.9980	0.0020	2.1541	0.0316	0.9842	0.0158
GARCH vs TGARCH	− 0.2184	0.8272	0.4136	0.5864	1.4450	0.1490	0.9255	0.0745	0.0310	0.9753	0.5123	0.4877	1.0436	0.2971	0.8515	0.1485
GARCH vs MGARCH	2.5565	0.0108	0.9946	0.0054	1.4604	0.1447	0.9277	0.0723	0.7818	0.4346	0.7827	0.2173	1.0315	0.3027	0.8486	0.1514
GARCH vs PGARCH	− 0.4029	0.6871	0.3436	0.6564	0.3784	0.7053	0.6474	0.3526	− 3.9597	0.0001	0.0000	1.0000	− 1.7911	0.0738	0.0369	0.9631
EGARCH vs TGARCH	− 4.8535	0.0000	0.0000	1.0000	− 0.2795	0.7800	0.3900	0.6100	− 3.3577	0.0008	0.0004	0.9996	− 0.9618	0.3365	0.1683	0.8317
EGARCH vs MGARCH	− 3.9551	0.0001	0.0000	1.0000	− 1.8251	0.0685	0.0342	0.9658	− 2.8046	0.0052	0.0026	0.9974	− 1.9776	0.0484	0.0242	0.9758
EGARCH vs PGARCH	− 3.5794	0.0004	0.0002	0.9998	− 0.7257	0.4683	0.2342	0.7658	− 5.1229	0.0000	0.0000	1.0000	− 2.2819	0.0228	0.0114	0.9886
TGARCH vs MGARCH	0.3195	0.7495	0.6253	0.3747	− 1.4181	0.1567	0.0783	0.9217	0.3417	0.7327	0.6336	0.3664	− 0.8380	0.4023	0.2012	0.7988
TGARCH vs PGARCH	− 0.1927	0.8472	0.4236	0.5764	− 0.9503	0.3423	0.1712	0.8288	− 4.0954	0.0000	0.0000	1.0000	− 2.5462	0.0111	0.0056	0.9944
MGARCH vs PGARCH	− 0.4957	0.6203	0.3102	0.6898	0.3523	0.7247	0.6376	0.3624	− 4.3316	0.0000	0.0000	1.0000	− 1.9407	0.0528	0.0264	0.9736
Recursive monthly									Rolling window monthly							
GARCH vs EGARCH	− 2.0439	0.0428	0.0214	0.9786	− 0.2824	0.7780	0.3890	0.6110	− 3.3505	0.0010	0.0005	0.9995	− 0.7561	0.4508	0.2254	0.7746
GARCH vs TGARCH	− 3.4247	0.0008	0.0004	0.9996	− 0.6268	0.5317	0.2659	0.7341	− 3.8377	0.0002	0.0001	0.9999	− 1.7552	0.0813	0.0407	0.9593
GARCH vs MGARCH	1.1143	0.2670	0.8665	0.1335	− 1.2213	0.2239	0.1120	0.8880	NA	NA	NA	NA	NA	NA	NA	NA
GARCH vs PGARCH	− 0.2165	0.8289	0.4145	0.5855	0.1851	0.8534	0.5733	0.4267	− 4.1044	0.0001	0.0000	1.0000	− 0.6650	0.5071	0.2535	0.7465
EGARCH vs TGARCH	− 1.7226	0.0871	0.0435	0.9565	− 0.8869	0.3766	0.1883	0.8117	− 1.5519	0.1228	0.0614	0.9386	− 1.5177	0.1312	0.0656	0.9344
EGARCH vs MGARCH	2.2638	0.0251	0.9875	0.0125	0.2105	0.8336	0.5832	0.4168	NA	NA	NA	NA	NA	NA	NA	NA
EGARCH vs PGARCH	3.6805	0.0003	0.9998	0.0002	1.5256	0.1293	0.9354	0.0646	− 0.1721	0.8636	0.4318	0.5682	0.5006	0.6174	0.6913	0.3087
TGARCH vs MGARCH	3.7699	0.0002	0.9999	0.0001	0.5696	0.5698	0.7151	0.2849	NA	NA	NA	NA	NA	NA	NA	NA
TGARCH vs PGARCH	3.8744	0.0002	0.9999	0.0001	1.3801	0.1697	0.9152	0.0848	1.5557	0.1219	0.9390	0.0610	1.6150	0.1085	0.9458	0.0542
MGARCH vs PGARCH	− 0.4011	0.6889	0.3445	0.6555	0.2793	0.7804	0.6098	0.3902	NA	NA	NA	NA	NA	NA	NA	NA

1. The columns labelled DM(A) and DM(S) contain t-statistic based on absolute and squared prediction errors, respectively. 2. The null hypothesis of DM-test is that of equal predictive ability of the two models; a significantly positive (negative) *t* statistics indicates the benchmark model is dominated by (dominates) the corresponding model

The conducted DM test results are mostly in line with the forecasting results, as can be seen from the tables below. A considerable portion of the pairwise comparisons show that the forecasting accuracy of one of the selected models is better based on the value of the error loss. Specifically, the daily results provide more significant values based on the absolute-error loss criteria for both recursive and rolling window methods. On the other hand, according to the daily results based on the squared-error loss, the NIKKEI, STI, SET, SSE and TAIEX indices cannot provide a definite forecasting accuracy between compared models due to the weakness of the DM test results.

The weekly results are more indecisive compared to the daily DM test results. The DM statistics for the NIKKEI and HSI Indices are less than 1.96, and therefore the zero hypothesis cannot be rejected. Thus, the observed difference between the forecasting performance of selected models is not significant and might be due to stochastic interference, which is in line with the findings of Burda and Bélisle (2019). The STI and KLCI indices also do not provide noteworthy test results based on the squared-error loss criterion. However, the remaining indices indicate similar results with empirical forecasting results for both the recursive and rolling window methods.

Finally, the forecasting comparison for the monthly return series reports significant forecasting accuracy for superior models, especially those based on the absolute-error loss. On the other hand, the DM statistics based on the squared-error criteria provide weaker results due to the smaller values for both recursive and rolling window methods: that is to say, the zero hypothesis cannot be rejected.

Summarizing the results listed in the following tables shows that the DM test results are highly consistent with the empirical volatility forecasts, indicating that the evaluations, of the forecasts are strong and accurate, as the outcomes are supported by the DM test statistics.

## Summary and conclusion

The present paper examines the volatility forecasting ability of the GARCH-type econometric models based on recursive and rolling window methods for ten Asian stock markets, inspired by the theoretical gap in model accuracy and the practical need for more comprehensive evidence for the selected markets and models. Five GARCH models are considered, namely GARCH, GARCH-M, EGARCH, TGARCH and PGARCH models where the first two represents symmetric and the remaining three represents asymmetric models. Daily, weekly and monthly return series data has been used and the evaluation of the forecasts are determined by using five different error statistics.

Based on the empirical analyses, GARCH-type models can appropriately adapt to the volatility behavior of Asian stock indices and provide a satisfactory degree of forecast accuracy in all selected time frames. The superiority of asymmetric models is more evident for higher time frames, while symmetric models tend to outperform in lower time frames. More precisely, the EGARCH model generates the most accurate volatility forecasts, closely followed by the TGARCH and PGARCH models for the daily and weekly frequencies, indicating that asymmetric specification of volatility dynamics needs to be taken into account: a finding which is in line with the study of Anggita (2020). This outcome also further implies that the asymmetric models might be more appropriate than the symmetric models when applying risk management strategies for Asian stock markets. This result is contradictory to Sharma et al. (2021), in which they argue that linear GARCH models are superior to non-linear. One potential explanation is that the present paper considers student’s t-distribution to accommodate fat tails and excess kurtosis, which reduces the chance of bias and the supports to capture of volatility asymmetries in the non-linear models. However, when it comes to monthly return series, the GARCH-M model gains more attention and the superiority of non-linear models decrease compared to higher time frames. Moreover, the use of three different frequencies not only implies that not just the ranking differs when applying various error statistics, but also shows how significantly it can differ. There is an important controversy over the fact that one error statistic suggests that a particular model is the best, while another error statistic suggests that the same model to be the worst. This highlights the importance of choosing a proper error statistic for the intended purpose of the forecast.

For a better visualization of the performance of the employed models and overall conclusion, Tables 19, 20 and 21 have been created. According to Table 19, the EGARCH model is clearly superior for both methods, followed by the TGARCH model. Performance records of the GARCH, PGARCH and GARCH-M models report double-digit numbers in terms of worst overall performance, while the EGARCH model does not report any numbers among the worst performers for either method. This makes the model is a clear winner and highlights the asymmetric specification of volatility dynamics in daily return series. Rolling window GARCH and GARCH-M models do not provide any accurate forecast values and are therefore the worst performers. These results are consistent with Awartani and Corradi (2005), Hansen and Lunde (2005), and Evans and McMillan (2007).

**Table 19:** Summary of performance ranking of the models for daily return series

Loss function	MAE		MAPE		RMSE		QLIKE		MSE		TOTAL	
Performance/models	Best	Worst	Best	Worst	Best	Worst	Best	Worst	Best	Worst	Best	Worst
Recursive GARCH	1	2	1	2	0	4	0	3	0	5	2	16
Rolling window GARCH	0	2	0	3	0	2	0	4	0	3	0	14
Recursive EGARCH	7	0	1	4	9	0	6	0	9	0	32	0
Rolling window EGARCH	10	0	4	0	6	0	7	0	6	0	33	0
Recursive TGARCH	0	1	7	0	1	1	2	0	1	0	11	2
Rolling window TGARCH	0	3	3	0	3	1	1	1	3	1	10	6
Recursive GARCH-M	2	0	0	4	0	1	1	3	0	1	3	9
Rolling window GARCH-M	0	0	0	7	0	4	0	2	0	2	0	15
Recursive PGARCH	0	7	1	0	0	4	1	4	0	4	2	19
Rolling window PGARCH	0	5	4	0	1	3	2	3	1	4	8	15

**Table 20:** Summary of performance ranking of the models for weekly return series

Loss function	MAE		MAPE		RMSE		QLIKE		MSE		TOTAL	
Performance/models	Best	Worst	Best	Worst	Best	Worst	Best	Worst	Best	Worst	Best	Worst
Recursive GARCH	1	1	1	1	0	3	3	4	0	2	5	11
Rolling window GARCH	0	3	2	1	0	1	1	0	0	1	3	6
Recursive EGARCH	6	0	6	3	5	1	0	1	5	1	22	6
Rolling window EGARCH	7	0	0	2	7	0	4	0	7	0	25	2
Recursive TGARCH	2	2	1	0	2	1	3	0	2	2	10	5
Rolling window TGARCH	2	2	6	1	2	1	2	1	2	1	14	6
Recursive GARCH-M	1	1	1	2	1	1	1	2	1	1	5	7
Rolling window GARCH-M	1	1	1	3	1	2	3	2	1	2	7	10
Recursive PGARCH	0	6	1	4	2	4	3	3	2	4	8	21
Rolling window PGARCH	0	4	1	3	0	6	0	7	0	6	1	26

**Table 21:** Summary of performance ranking of the models for monthly return series

Loss function	MAE		MAPE		RMSE		QLIKE		MSE		TOTAL	
Performance/models	Best	Worst	Best	Worst	Best	Worst	Best	Worst	Best	Worst	Best	Worst
Recursive GARCH	2	0	3	2	3	0	4	0	3	0	15	2
Rolling window GARCH	4	1	4	3	2	0	5	0	2	0	17	4
Recursive EGARCH	2	3	2	3	3	0	1	3	4	0	12	9
Rolling window EGARCH	2	1	3	2	3	2	2	1	3	1	13	7
Recursive TGARCH	0	2	1	2	0	3	0	3	0	3	1	13
Rolling window TGARCH	1	0	0	2	2	1	1	1	2	2	6	6
Recursive GARCH-M	5	0	1	3	2	0	5	2	1	0	14	5
Rolling window GARCH-M	1	6	1	1	1	3	0	5	1	3	4	18
Recursive PGARCH	1	5	3	0	2	7	0	2	2	7	8	21
Rolling window PGARCH	2	2	2	2	2	4	2	3	2	4	10	15

The “Best” and the “Worst” columns in Tables 19, 20 and 21 indicate the number of times that the selected model is ranked as the best or the worst based on the corresponding loss function. The “TOTAL” column summarizes the total number of times a forecasting model is ranked as the best (worst)

Table 20 indicates that the EGARCH and TGARCH models provide the lowest error statistics in total compared to other models, which make them the best performers for weekly return series. Surprisingly, the PGARCH model becomes the worst forecasting model based on the reported values. The GARCH and GARCH-M models increase in forecasting powers compared to daily results, which suggests that, symmetric models should be considered for a better risk management purposes in selected Asian markets for weekly returns series. The results are in partially in line with those of Ng and McAleer (2004), Liu et al. (2009), Mwita and Nassiuma (2015), and Sharma (2016).

Based on the reported values by Table 21, EGARCH still provides a strong forecast performance record compared to its asymmetric counterparts, while GARCH seems to be the best forecasting model for monthly return series. This may be due to the reducing asymmetric volatility dynamics in the lower frequencies. Furthermore, the GARCH-M model indicates mixed results, which seem to be penalized more heavily by the rolling window method, while the recursive method put it among the best performers. The PGARCH model is the clear loser, followed by the TGARCH model. These findings are in line with Balaban (2004), but contradicts with Atoi (2014), which recommends the PGARCH model as a best performer.

Through the analyses above, the following conclusion can be drawn.

- Symmetric and asymmetric GARCH models can be applied to Asian stock markets. Although these models were developed and widely used in the process of researching Western financial markets, it does not obstruct the use of them in emerging or developed Asian financial markets.
- In terms of the time series perspective, the volatility behavior of Asian markets indicates considerable clustering and time-varying events. This is more evident during the turbulent times, such as the 1997–1998 Asian crisis and the 2008 US subprime crisis, due to the information shock on the markets reflecting the phenomenon whereby large changes tend to be followed by large changes, of either sign, and small changes tend to be followed by small changes.
- Given the level of risk associated with investment in stock markets, day traders, investors, financial analysts and empirical finance professionals should consider alternative error distributions while specifying predictive volatility model as less contributing error distributions implies incorrect specification, which could lead to loss of efficiency in the model. Investors should also not ignore the impact of news while forming expectations on investments.
- Frequency of the data and choice of forecast method have a strong effect on model performance, and therefore, depending on the investment perspective and risk sensitivity, the correct method and time frames should be applied.

The out-of-sample performance of the compared volatility models in terms of the different loss functions based on the three data sets, thus suggests a bit of a challenge. It is far from evident which of the specific conditional volatility models outperforms the other. First, the ranking of models based on a specific loss function differs for the three data sets. Secondly, for the selected markets the best and worst model depends heavily on which loss function is used. To answer which model has the best out-of-sample performance, one must first consider the specific data set used and then which loss function to use as the criteria.

The main limitation of this study is data availability, especially for the higher frequency of data in the emerging countries of Asia. Further research could explore a wider sample of financial markets—Vietnam, India, Russia and other countries in Asia—with more up-to-date data considering the recent COVID-19 crisis and the war between Ukraine and Russia. This would explore how the news information impacts volatility behavior across stock markets of Asia. Another agenda for future research could include a wider set of GARCH family models to test and estimate the forecasting accuracy of a wider sample.

## Data Availability

The data that support the findings of this study are available from Bloomberg database upon subscription.

## Code availability

The codes that support the findings of this study are available from the author on request.
